# Synthesis, Hydrolysis, and Protonation-Promoted Intramolecular Reductive Breakdown of Potential NRTIs: Stavudine α-*P*-Borano-γ-*P*-*N*-l-tryptophanyltriphosphates

**DOI:** 10.3390/molecules201018808

**Published:** 2015-10-16

**Authors:** Zhihong Xu, Barbara Ramsay Shaw

**Affiliations:** Department of Chemistry, Duke University, Durham, NC 27708, USA; E-Mail: barbara.r.shaw@duke.edu

**Keywords:** boron chemistry, pronucleotide, d4T boranotriphosphate analog, stability, mechanism, intramolecular reduction

## Abstract

Phosphorus-modified prodrugs of dideoxynucleoside triphosphates (ddNTPs) have shown promise as pronucleotide strategies for improving antiviral activity compared to their parent dideoxynucleosides. Borane modified NTPs offer a promising choice as nucleoside/nucleotide reverse transcriptase inhibitors (NRTIs). However, the availability of α-*P*-borano-γ-*P-*substituted NTP analogs remains limited due to challenges with synthesis and purification. Here, we report the chemical synthesis and stability of a new potential class of NRTI prodrugs: stavudine (d4T) 5′-α-*P*-borano-γ-*P*-*N*-l-tryptophanyltriphosphates. One-pot synthesis of these compounds was achieved via a modified cyclic trimetaphosphate approach. Pure *R*p and *S*p diastereomers were obtained after HPLC separation. Based on LC-MS analysis, we report degradation pathways, half-lives (5–36 days) and mechanisms arising from structural differences to generate the corresponding borano tri- and di-phosphates, and H-phosphonate, via several parallel routes in buffer at physiologically relevant pH and temperature. Here, the major hydrolysis products, d4T α-*P*-boranotriphosphate *R*p and *S*p isomers, were isolated by HPLC and identified with spectral data. We first propose that one of the major degradation products, d4T H-phosphonate, is generated from the d4T pronucleotides via a protonation-promoted intramolecular reduction followed by a second step nucleophilic attack. This report could provide valuable information for pronucleotide-based drug design in terms of selective release of target nucleotides.

## 1. Introduction

Naturally occurring or chemically synthesized nucleoside analogs have been used as anti-viral and anti-tumor drugs for decades. Mechanistic studies show that a dideoxynucleoside (ddN) must be activated in a series of phosphorylation reactions to the corresponding 5′-triphosphate (ddNTP) active form, which is then incorporated as a chain terminator by a viral reverse transcriptase into the viral DNA strand [1]. Several nucleoside analogs such as AZT and d4T are widely used in the treatment of AIDS patients, but major concerns such as drug toxicity, ineffectiveness and resistance resulting from inefficient phosphorylation by intrinsic kinases after cell penetration are often associated. To overcome such disadvantages, various nucleotides and other modified analogs have been studied as potential nucleoside/nucleotide reverse transcriptase inhibitors (NRTIs). Prodrug approaches can be effective strategies in new drug discovery and development, which involve modifying the drug to enable it to better enter the cell, and converting the drug to its active form through either chemical and/or enzymatic activation. The pronucleotide approach [2,3,4,5,6,7] allows the bypass of one or more phosphorylation steps with the prospect of also improving the stability, bioavailability, and efficacy of the parent drug.

The delivery of a number of nucleoside monophosphates by pronucleotide approaches has improved antiviral activity and decreased cytotoxicity compared to their parent nucleosides [8,9]. McGuigan *et al.* [10,11] reported that phosphoramidate pronucleotides led to a significant enhancement of potency against HIV and HBV. Recently, a 1′-cyano-2′-*C*-methyl 4-aza-7,9-dideaza adenosine phosphoramidate analog was reported by Cho and co-workers [12] to be a potent, selective inhibitor of HCV polymerase NS5B with high liver triphosphate loading, although its clinical utility was limited by high intra-/interpatient pharmacokinetic and pharmacodynamic variability. The P–N bond of nucleoside phosphoramidates can be cleaved intracellularly by intrinsic enzymes such as histidine triad nucleotide-binding protein 1 (Hint 1) [9]. Although amino acid phosphoramidates and other prodrugs of nucleoside monophosphates have shown promise in the pronucleotide strategy [8,9,10,11,12,13,14,15,16,17], further optimization of this type of prodrug is needed. For some clinically used ddNs or other lead nucleosides, the rate limiting steps may involve phosphorylation of the nucleoside analog from its monophosphate to the corresponding diphosphate, and/or from the diphosphate to the active triphosphate form. In such cases, nucleoside mono- and/or diphosphate prodrugs may not have prominent advantages over parent nucleoside analogs themselves.

Because a borane group (BH_3_) is isoelectronic and isosteric with oxygen, yet more hydrophobic, a non-bridging α-P oxygen can be replaced by a BH_3_ to give α-*P*-BH_3_ nucleotide analogs [18,19]. The *P*-boranophosphates are near perfect mimics of natural nucleic acids for reading and writing genetic information with high yield and accuracy [20,21]. The α-*P*-borano ddNTPs are better chain terminators for drug-resistant viral reverse transcriptases (RTs) than parent ddNTPs [22], and the presence of the α-*P*-borano group leads to increased stability towards 3′ exonuclease [23] and pyrophosphorolytic repair of a dideoxy nucleoside blocked DNA chain [24], as well as enhanced lipophilicity and other unique properties [25,26,27]. In human cells, the metabolism of d4T to its monophosphate by thymidine kinase is the bottle-neck. However, although a 10-fold enhancement of the phosphorylation by nucleoside diphosphate kinase for the *R*p isomer of ddN α-*P*-boranodiphosphates (ddNDPαBs) relative to AZTDP or d4TDP has been reported [25], the catalytic efficiency for ddNDPαBs is still 1000-fold less than for the parent TDP. Therefore, we believe analogs of nucleoside triphosphate might provide a better choice.

The possibility of designing nucleoside triphosphate (NTP) mimics that entirely bypass cellular phosphorylation has been a subject in the nucleoside analog research area for decades [2]. However, studies of certain modifications of nucleoside triphosphates at the γ-phosphate have been limited [28,29], due to challenges in chemical synthesis, purification, and prodrug stability. In Wang *et al.*’s report [28], AZT 5′-α-*P*-borano-β,γ-difluoromethylene-γ-*P*-*O*-substituted triphosphate mimics were synthesized from the corresponding triphosphates by direct methylation of a triphosphate with methyl trifluoromethanesulfonate (*R*p/*S*p mixture yield: 2.3%) or condensation of a DCC activated triphosphate with a phenoxide ion (*R*p/*S*p mixture yield: 32.0%). However, pure *R*p and *S*p diastereomers were not successfully obtained after their efforts towards reverse-phase HPLC separation. The final samples used for their biological property studies were 94% or 84.6% *R*p/*S*p mixtures along with other unknown impurities. Even though rather impure, their AZT 5′-α-*P*-boranotriphosphate analog mixtures still exhibited strong inhibitory effects against HIV-1 RT [28]. In comparison with AZTTP, TTP and ATP, Wang *et al.* also reported much longer half-lives of AZT 5′-α-*P*-borano and β,γ-bridge modified triphosphates in fetal calf serum and CEM cell extract at 37 °C. Therefore, we have explored herein a more efficient way to obtain pure stereoisomers of α-*P*-borano-γ-*P-N-*substituted triphosphate mimics for future biological and pharmaceutical investigations.

A cyclic trimetaphosphate approach was first reported by Ludwig and Eckstein for the synthesis of nucleoside triphosphates and α-*P*-thiotriphosphates [30]. Later, this protocol was modified by us and other groups for synthesis of a variety of nucleoside α-*P*-borano-, thio- or seleno- triphosphates [28,31,32,33] or diphosphates [34], tryptophanyltriphosphoramidates [35], ddN triphosphoramidates [28], and nucleoside tetraphosphate analogs [36,37,38,39]. We observed that by replacing ethylenediamine (in the synthesis of nucleoside boranodiphosphates [34]) with l-tryptophan methyl ester [35] as a trimetaphosphate ring opening reagent, a number of nucleoside boranotriphosphate prodrugs could be conveniently made. In our first synthesis [35] of α-*P*-borano- and thio- modified nucleoside γ-*P-N*-substituted triphosphate mimics via cyclic trimetaphosphate intermediates, the isolation of the respective *R*p and *S*p diastereomers in their triethylammonium salt (TEA salt) form was successfully achieved after HPLC purification.

**Figure 1:**
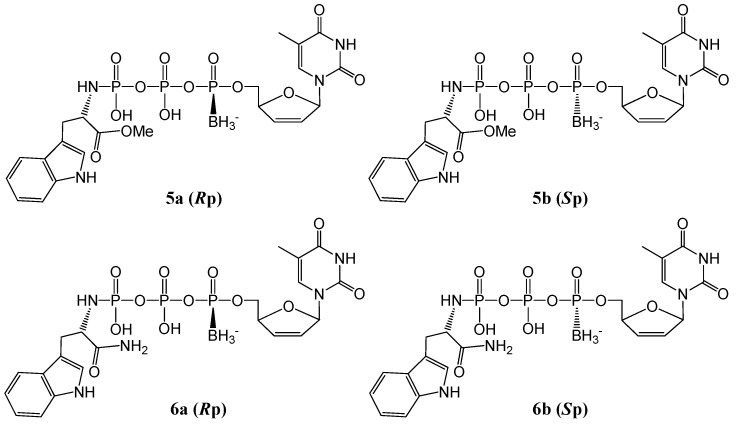
Structures of d4T α-*P*-borano-γ-*P-N*-l-tryptophanyltriphosphates (**5a**, **5b**, **6a**, **6b**).

Here, we use stavudine (d4T) 5′-α-*P*-borano-γ-*P*-*N*-l-tryptophanyltriphosphates (**5a**, **5b**, **6a**, and **6b**, Figure 1) as ddN triphosphoramidate templates for the design of ddN boranophosphates conjugated with tryptophan as potential new NRTI prodrugs. We synthesized the phosphoramidate mimics because of the known instability of a P–N bond in cellular media, and thus chose tryptophan as the masking group to improve the lipophilicity; also tryptophan is a non-toxic natural amino acid with ideal UV absorption (enabling detection). It is important to identify possible side products remaining from unsuccessful purification, and examine possible degradation products arising from a continuous degradation of the final products during sample handling in aqueous solutions. In this article, the synthesis, isolation, and stabilities of the title compounds in buffer are described and discussed in detail. Further, the prodrug degradation pathways and mechanisms imparted by the structural differences are proposed for the first time.

## 2. Results and Discussion

### 2.1. Synthesis and Isolation

**Scheme 1:**
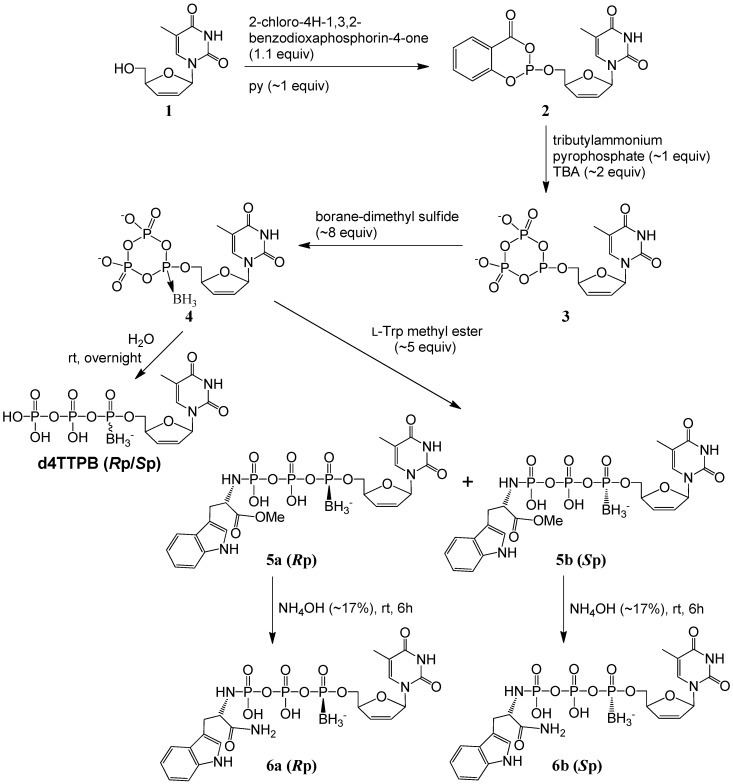
One-pot synthesis of d4T α-*P*-borano-γ-*P*-*N*-l-tryptophanyltriphosphate **5a**, **5b**, **6a**, and **6b** stereoisomers by a modified cyclic trimetaphosphate approach.

In our modified cyclic trimetaphosphate approach (Scheme 1) for the synthesis of α-*P*-boranotriphosphate prodrugs, d4T (**1**) was first phosphitylated to **2**, followed by treatment of the reactive phosphite with tributylammonium pyrophosphate to yield a cyclic trimetaphosphite intermediate **3**, following the Ludwig-Eckstein procedure. After a boranation step to form **4**, l-tryptophan methyl ester was used as a nucleophile to open the trimetaphosphate ring of **4** at room temperature (rt) in the subsequent step. (Excess triethylamine had been used in advance to free the α-amino group of l-tryptophan methyl ester·HCl). The nucleophilic attack on either one of the two β-P positions in the ring of **4** by the α-amino group of tryptophan methyl ester proceeded readily to give the title compound d4T 5′-α-*P*-borano-γ-*P*-*N*-l-TrpOMe triphosphates **5** in ~30% isolated yields by UV, ~15% for each isomer **5a** or **5b** (see Experimental Section for isolation). After the reactions to generate **5** were finished, the reaction mixture was poured into cold diluted ammonium hydroxide (NH_4_OH) solution (in an icy water bath) right before solvent evaporation. By contrast, when water was directly added, most compounds **5** decomposed, probably due to the increased acidity. This was in agreement with the reports [29,40] that the P–N bond of nucleoside *N*-alkylphosphoramidates was chemically cleaved in acidic solutions.

The introduction of a borane group on the α-P generates a pair of diastereomers (*i.e.*, *R*p and *S*p isomers), which were resolved by HPLC, as shown in Table 1. Since it has been well established that (a) deoxyribonucleoside boranotriphosphates can be incorporated into DNA chains by DNA polymerases, and the isomer first eluted from HPLC was incorporated much more efficiently than the isomer in the second eluted peak [23,41]; (b) the *R*p analogs of ddN boranotriphosphates are the NRTI active isomers [24]; (c) the first eluting ddNTP analogs from HPLC isolation showed more potent activities (than the second eluting diastereomers) and therefore were assigned as the *R*p isomers [28]; and (d) the assignment of both P-diastereomers of ATP analogs was supported by NMR [42], we thus deduce that the first (**5a**) and second (**5b**) eluting diastereomers are the *R*p and *S*p isomers, respectively. For further confirmation, here the specific isomers of the major hydrolysis products d4T 5′-α-*P*-boranotriphosphates (d4TTPαB, **7a** and **7b**, generated from **5a** and **5b** γ-P–N bond cleavage) were isolated from pools of the respective incubation solutions using HPLC (1% acetonitrile (ACN) for 40 min followed by 1%–20% ACN over an additional 40 min in 20 mM triethylammonium acetate (TEAA) buffer). Isolated *R*p and *S*p isomers of d4TTPαB were identified using UV, LC-MS, and NMR (see Experimental Section for spectroscopic data, and Supporting Information (SI) Figures S19–S22 for spectra), in combination with the spiking of the synthesized d4TTPαB *R*p/*S*p mixture (see the Experimental Section for synthesis) with the pure isolated *R*p or *S*p isomer followed by LC-MS analysis (see Supplementary Figure S6).

Further treatment of each isomer of **5** with ~17% NH_4_OH for 6 h produced the respective amide analog **6a** or **6b** (~33% aminolysis conversion) in an overall 5%–7% isolated yield (by UV). For the base treatment of **5** to generate **6**, longer time produced more **6** but also more other degradation products.

For all desired sample fractions after HPLC purification, acetonitrile was evaporated at low temperature (<35 °C), and the respective isomer water solution was lyophilized. All pure compounds were obtained in their TEA salt form. As expected in the HPLC profile, l-tryptophan methyl ester mimics (**5a** and **5b**) had longer retention times than the corresponding amide analogs (**6a** and **6b**). Since **6a** and **6b** were the aminolysis products from the corresponding **5a** (*R*p) and **5b** (*S*p) isomers (with structural change on the l-Trp methyl ester moiety only, thus stereo specificity at the α-P was retained), and **6a** had a shorter retention time than **6b**, it follows that **6a** and **6b** should be the *R*p and *S*p isomers, respectively. After separation, the purity and identity of all isomers were verified by HPLC, NMR and HRMS. Detailed spectroscopic data and NMR scans of the isomers are given in the Experimental Section and Supplementary Figures S7–S18. The overall isolated yields and HPLC profile of the title compounds are listed in Table 1.

**Table 1:** Overall isolated yields and HPLC profile of d4T α-*P*-borano-γ-*P*-*N*-l-tryptophanyltriphosphate *R*p and *S*p Isomers.

d4TTPαB Analog	MW by LC-MS	% Yield ^a^	HPLC ^b^	
			ACN (%)	*t*_R_ (min)
**5a**	661	~30	15	7.9
**5b**	661		15	11
**6a**	646	~12	15	4.1
**6b**	646		15	4.4

^a^ Isolated yield calculated in percentage by UV; ^b^ Eluted at 1 mL/min with isocratic condition of 15% ACN/10 mM pH 7 TEAA. Column: Delta-Pak™ C18 (Waters, Milford, MA, USA), 15 µm, 3.9 mm × 300 mm. *t*_R_: retention time.

### 2.2. Stability Studies

Property studies reported here focus on chemical stability of the title compounds in pH 7.4 Tris buffer free of enzyme at 37 °C, in order to identify possible decomposition products during sample purification and handling in aqueous solutions. The title compounds were kept frozen before analysis by LC-MS, and the data points were collected at different incubation times. Our results indicated that these compounds are reasonably stable in pH 7.4 Tris buffer at 37 °C; half-lives (5–36 days) are shown in Table 2. Within expectation, the stability varied with the structural modifications. For d4T α-*P*-boranotriphosphate mimics, the l-tryptophan methyl ester analogs (**5a** and **5b**) were more stable than their corresponding amide analogs (**6a** and **6b**) in the tested buffer (Table 2). Interestingly, **5a** was more stable than **5b**, while **6a** was less stable than **6b**. Further, major nucleotides released from the title compounds were identified by mass to be a d4T α-*P*-boranotriphosphate (d4TTPαB, 7, *m*/*z* 461) isomer and d4T H-phosphonate (d4TH-P, 8, *m*/*z* 287) for 5, in contrast to a d4T α-*P*-boranodiphosphate (d4TDPαB, 9, *m*/*z* 381) isomer and d4TH-P for 6, which indicated that different degradation pathways arise from structural differences. Structures of major degradation products and example LC-MS analyses are included in the Supplementary Chart S1 and Figures S3–S5. The percentages of major degradation products and intact prodrugs after 1.5 and/or 7.7 day incubation are also listed here in Table 2.

**Scheme 2:**
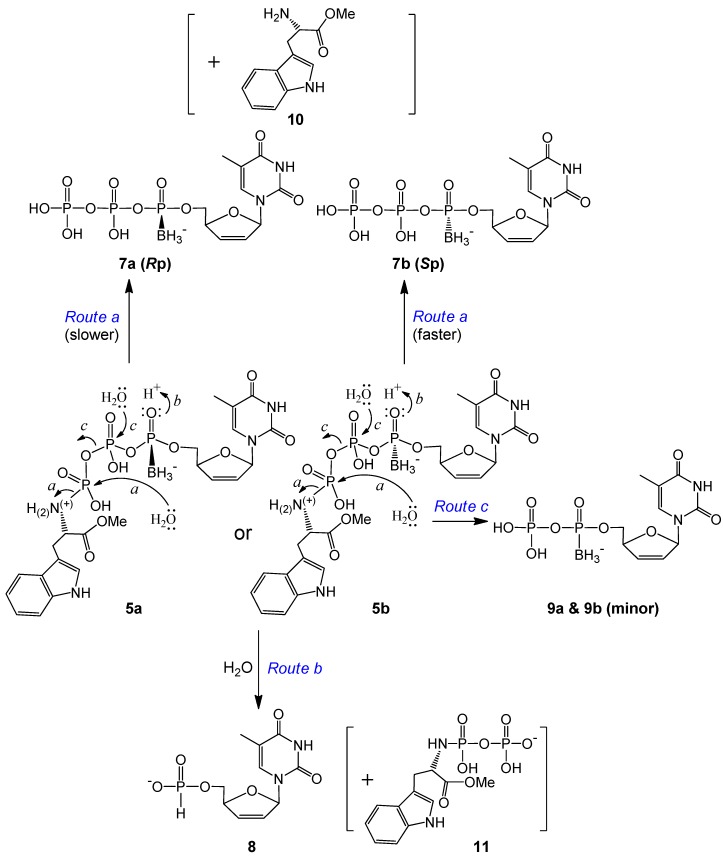
Proposed degradation pathways of d4T 5′-α-*P*-borano-γ-*P*-*N*-l-TrpOMe triphosphates **5a** and **5b** (α-P stereoisomers) in pH 7.4 Tris buffer at 37 °C.

The d4T 5′-α-*P*-borano-γ-*P*-*N*-l-TrpOMe triphosphates **5a** (*R*p) and **5b** (*S*p) yielded mainly the corresponding d4TTPαB (**7a** or **7b**) and d4TH-P (**8**), along with a small quantity of d4TDPαB (**9a** or **9b**) and the masking moiety, l-tryptophan methyl ester (TrpOMe, **10**) (Scheme 2 and example analyses in Figures S3 and S4 of SI). Over time, the amount of **10** detected (M^−^ 217) was unpredictable by the LC, probably due to further degradation of this compound in the incubation solution. Because of the lability of the P–N bond, especially facilitated by the nitrogen protonation, the γ-P is prone to the nucleophilic attack by water, which generates the major hydrolysis product d4TTPαB *R*p or *S*p isomer, and releases the masking group via path *a* (hydrolysis), as proposed in Scheme 2. Over time, more d4TTPαB isomers (**7a** and **7b**) were generated compared to d4TH-P from 5a and 5b. Because the leaving group (l-TrpOMe) is pointing rear, we propose that the nucleophile (H_2_O) attacks the γ-P from the front side. Therefore, for the *R*p isomer, the incoming nucleophile (H_2_O) and the borane group electrons might repel with each other, which makes this hydrolysis process slower; while in the case of the *S*p isomer, the borane group is on the same side with the large leaving group in the back, so the front side nucleophilic attack by water is not hindered, and the leaving of the large l-TrpOMe moiety further relieves the steric strain. Therefore, **5a** produced considerably less d4TTPαB *R*p (**7a**) than did **5b** for d4TTPαB *S*p (**7b**) during the same incubation times (Table 2). Only minor amounts of d4TDPαB were produced by nucleophilic attack of water on the β-P via *Route c* and further hydrolysis of d4TTPαB.

**Table 2:** Stability studies of d4T α-*P*-borano-γ-*P*-*N*-l-tryptophanyltriphosphates by LC-MS in pH 7.4 Tris buffer at 37 °C.

d4TTPαB Analog	*t*_1/2_ (d)	Major Degradation Products (a%, b% *)	% of Intact Molecule ^ǂ^
**5a**	35.9	d4TTPαB *R*p **7a** (1.8%, 7.1%)	~81%
		d4TH-P **8** (2.3%, 2.8%)	
**5b**	26.6	d4TTPαB *S*p **7b** (8.8, 16%)	~68%
		d4TH-P **8** (6.7%, 7.9%)	
**6a**	5.0	d4TDPαB *R*p **9a** (>14%, >39%)	~24%
		d4TH-P **8** (2.6%, 4.9%)	
**6b**	6.4	d4TDPαB *S*p **9b** (~13%, ~31%)	~30%
		d4TH-P **8** (2.6%, ~8%)	

* a% = LC peak area% after ~1.5 day incubation; b% = LC peak area% after ~7.7 day incubation. **^ǂ^** LC peak area% of **5a**, **5b**, **6a**, or **6b** remaining after ~7.7 day incubation. LC-MS: Eluted at 0.3 mL/min with 0%–25% B in 25 min (Solvent A: 10 mM pH 7 TEAA; Solvent B: 100% ACN). Column: Eclipse XDB C-18, 2.1 mm × 50 mm ZORBAX, 3.5 μm (Thomas Scientific, Swedesboro, NJ, USA). UV detection at 272–288 nm. MS: Negative ion detection.

**Scheme 3:**
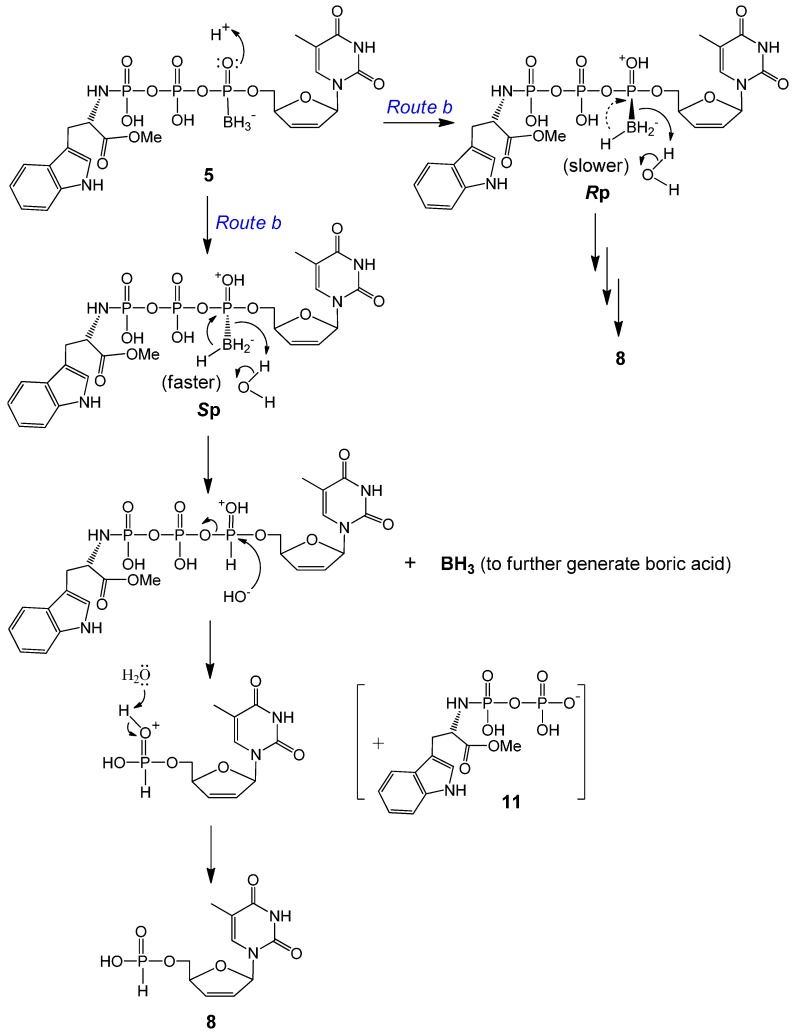
Proposed protonation-promoted intramolecular reductive deboranation followed by intermolecular hydrolysis to generate d4TH-P **8** from **5a** or **5b** (*Route b* in Scheme 2).

Direct attack by water on the α-P of **5** to generate d4T boranomonophosphate (d4TMPB) was not supported by our LC-MS data; instead, d4TH-P **8** was detected as one of the major degradation products in our LC-MS experiments (Table 2). As the electron distribution is more polarized toward oxygen on the boranated phosphorus [19,43], we propose that the non-bridged oxygen on the α-P is easily protonated, and this protonation effectively promotes an intramolecular nucleophilic attack by a hydride from the borane group (BH_3_), which leads to d4TH-P **8** formation (intramolecular reduction via *Route b*, Scheme 2). This reductive deboranation process, followed by an intermolecular hydrolysis step, is further outlined in Scheme 3. It was noticeable that the *R*p isomer generated considerably less **8** than did the *S*p isomer during the same incubation times (Table 2). We propose that for the *R*p isomer, the borane group is pointing front, enabling hydride attack on the α-P from the backside (the same side with the big moiety TrpOMe); therefore, the reduction process is slower. Whereas for the *S*p isomer, the hydride attacks the α-P from the front side, and the deboranation step further relieves the steric interaction between the borane and TrpOMe moieties in the rear, and hence faster. Only a small amount of compound **11** (TrpOMeDP, M^−^ 377) was detectable, likely due to lability of the P–N bond.

**Scheme 4:**
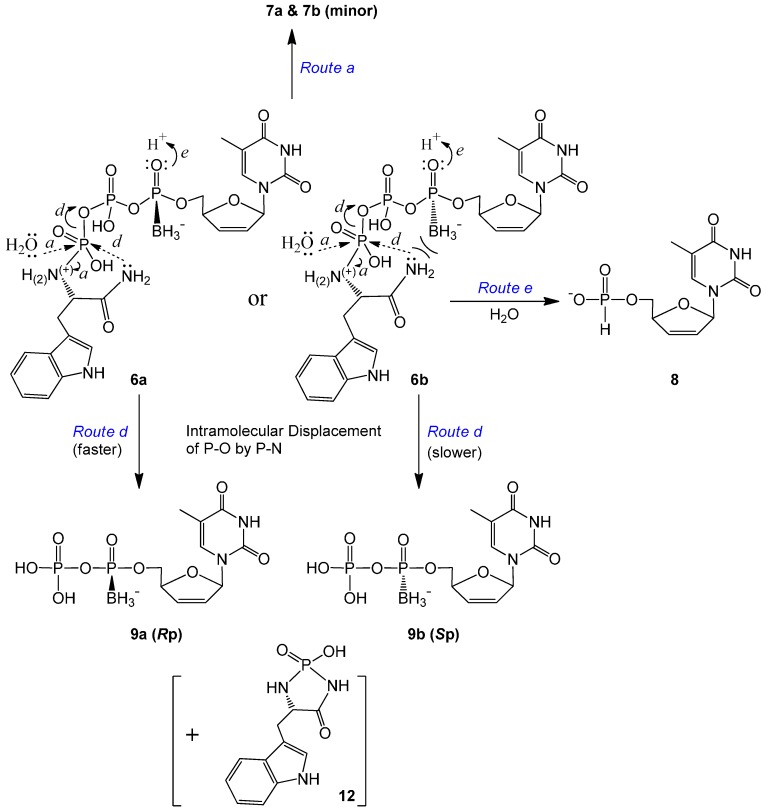
Proposed degradation pathways of d4T 5′-α-*P*-borano-γ-*P*-*N*-l-TrpNH_2_ triphosphates **6a** and **6b** (α-P stereoisomers) in pH 7.4 Tris buffer at 37 °C.

Compared to **5**, the degradation rate of the compounds with an amide bond on the tryptophan moiety (**6a** and **6b**) increased noticeably. For each isomer, only minor triphosphate product d4TTPαB was observed (via *Route a* in Scheme 4). The major degradation product here was the corresponding diphosphate d4TDPαB (**9a** or **9b**, which might be further phosphorylated in cells), whereas d4TH-P **8** was generated at a much smaller amount compared to **9** (example analyses: Figures S3 and S5 in the SI). Since the replacement of OMe (in **5**) by NH_2_ (in **6**) on the Trp carbonyl group led to the generation of a large quantity of d4TDPαB at a noticeably faster rate, we propose that the lone pair electrons on this amino group, although less reactive compared to other primary amines because of the amide resonance structure, are sufficiently nucleophilic to perform the intramolecular attack on the nearby γ-P (but not the α-P, where stereo specificity is retained) to generate the diphosphate (leaving group/degradation product) and thereby form a tryptophanyl cyclomonophosphorodiamidate **12** (difficult to observe due to lability), as shown in Scheme 4, *Route d*. This is in agreement with our earlier report of using ethylenediamine to synthesize nucleoside diphosphate analogs via an intramolecular nucleophilic attack [34]. Because the TrpNH_2_ masking group in the pronucleotides is pointing rear, the amino group should attack the γ-P from the backside. For the *R*p isomer (**6a**), the lone pair electrons of N are on the opposite side from the borane group, hence there is no strong electronic repulsion in this case; while for the *S*p isomer (**6b**), the interaction of the nucleophile and the borane group (on the same side) may hinder the nucleophilic attack. Therefore, the γ-P of **6a** (*R*p) should be more prone to the intramolecular nucleophilic attack, resulting in less stability in comparison to **6b** (*S*p) (Table 2).

**Scheme 5:**
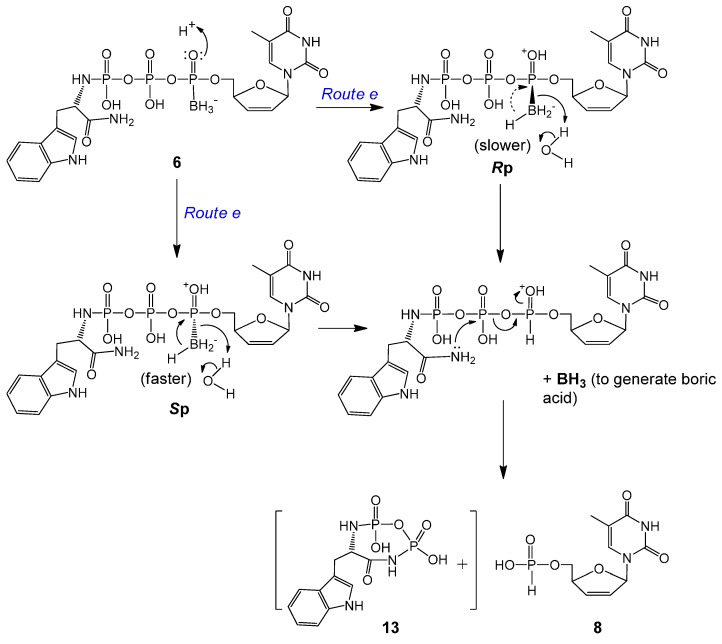
Proposed protonation-promoted intramolecular reductive deboranation followed by intramolecular P–O bond displacement by P–N bond to generate d4TH-P **8** from **6a** or **6b** (*Route e* in Scheme 4).

Within expectation, d4TH-P **8** was also generated from **6a** and **6b** during incubation (Scheme 4). However, no analog of compound **11** (TrpNH_2_DP, MW 362, replacement of OMe with NH_2_) was observed. Instead, a small peak with *m*/*z* 368 (345 + Na^+^) was detectable. We propose that although **8** (from **6** via *Route e*) is also generated through an intramolecular reduction followed by a nucleophilic attack, the second step nucleophile is the intramolecular amino group of the amide that attacks the β-P of **6** (but not the HO^−^ or water from the solution attack on the α-P), thereby resulting in the release of a tryptophanyl cyclodiphosphorodiamidate analog **13** (MW 345, small quantity detected due to lability), as further shown in Scheme 5. Although **6b** (*S*p, TrpNH_2_ and BH_3_ on the same side) degraded to d4TH-P at a rate faster than **6a** (*R*p), the formation of the corresponding d4TDPαB dominates the overall stability of the pronucleotides (**6b** more stable than **6a**, Table 2). Since more d4TDPαB (than d4THP) was generated from compounds **6**, we propose that the intramolecular displacement of the P–O bond with the P–N bond via nucleophilic attack by the amino group (of the amide) on the γ-P (*Route d*) is faster than that on the β-P which follows the protonation-promoted intramolecular reduction step (*Route e*).

## 3. Experimental Section

### 3.1. General Information

Chemical reagents and solvents were purchased from Sigma-Aldrich (St. Louis, MO, USA) and Fisher Scientific (Pittsburgh, PA, USA), unless otherwise indicated. Reactions were performed under an argon atmosphere at rt unless specified otherwise. For reaction mixtures, a Varian (Palo Alto, CA, USA) Inova-400 spectrometer was used to record ^31^P-NMR spectra at 162 MHz with broad band decoupling and reported in ppm downfield from the internal Varian 0 ppm standard. After purification, d4T boranotriphosphate analog NMR data were obtained on a Varian Inova-500 at 500 MHz for ^1^H- and 202 MHz for ^31^P- in D_2_O at 25 °C or 2 °C, or a Varian Inova-400 spectrometer at 400 MHz for ^1^H- and 162 MHz for ^31^P- in D_2_O at 25 °C, with chemical shift in ppm relative to 85% H_3_PO_4_ as an external reference. Ion-exchange chromatography was performed on an ISCO (Lincoln, NE, USA) system equipped with an anion exchanger QA-52 quaternary ammonium cellulose (Whatman, Marlborough, MA, USA) packed into a 1.5 cm × 30 cm glass column. 2 M TEAA (pH 7, Glen Research, Sterling, VA, USA) was diluted to 10 mM for analytical HPLC and 20 mM for preparative HPLC, unless specified otherwise. Analytical HPLC was performed on a Varian Star #1 system (Waters Delta-Pak™ C18 Column, 15 μm, 3.9 mm × 300 mm) with UV detection. Preparative HPLC was performed on a Waters™ Delta 600E system (XTerra Prep RP-18 Column, 5 μm, 10 mm × 150 mm) with a 996 photodiode array UV detector. Ultraviolet (UV) measurements were performed with a Varian Cary 100 Bio UV-Visible spectrometer, controlled by the Cary WinUV Analysis package. The final yields of the synthesized compounds were determined by UV absorption at their maximum absorbance wavelengths indicated in the spectroscopic data section. Compound molecular weight and the analysis of incubation mixtures were measured using the electrospray ionization mass spectrometer (ESI-MS) with an Agilent (Santa Clara, CA, USA) 1100 Series Liquid Chromatograph/Mass Selective Detector (LC/MSD) system. High resolution mass spectra were recorded on a JEOL JMS-SX-102 spectrometer (FABMS, Jeol Co. Ltd, Tokyo, Japan) or an Agilent G6224 LCMS-TOF with a Series 1200 HPLC by Dr. George R. Dubay in the Duke Chemistry Department. Tris buffer (pH 7.4) was prepared by mixing Tris acid and Tris base using Milli-Q deionized (DI) water, and filtered (syringe filter, 0.2 μm pore size, 13 mm diameter, Whatman GD/X). A Beckman (Brea, CA, USA) Φ34 pH meter and a Beckman Futura™ gel-filled combination pH electrode (Ag/AgCl) were used to measure pH values of buffers.

### 3.2. Procedures for the Synthesis and Isolation of ***5a***, ***5b***, ***6a***, ***6b***, and d4T α-P-Boranotriphosphates

Dideoxynucleoside d4T **1** was dried in a desiccator in vacuo at rt (22 °C) over P_2_O_5_ for ~12 h before use. Commercially available l-tryptophan methyl ester (HCl salt form) was treated with excess triethylamine and filtered before drying over vacuum overnight. Tri-*n*-butylamine (TBA) and tributylammonium pyrophosphate were dried with molecular sieves (A4). Other chemicals were used as received. As shown in Scheme 1, phosphitylation of d4T (0.8 mmol) was carried out in 1.5 mL anhydrous ACN and pyridine (~1 equiv) with ~1.1 equiv 2-chloro-4*H*-1,3,2-benzodioxaphosporin-4-one for 10 min to yield two diastereomers of d4T-5′-(4*H*-1,3,2-benzodioxaphosporin-4-one) **2**, as indicated by the appearance of a doublet at ~127 ppm in the ^31^P-NMR of the reaction mixture in deuterated ACN. Tributylammonium pyrophosphate (~1 equiv) in ACN was then added and stirred for ~20 min in the presence of TBA (~2 equiv) to form a d4T *P*^2^,*P*^3^-dioxo-*P*^1^-cyclotriphosphite **3**, which was indicated by the upfield shift from ~127 ppm to a triplet at 108 ppm for trivalent α-P and a doublet at around −16 ppm for pentavalent phosphorus signals. The α-P boranated compound **4** was obtained by the treatment of **3** with borane-dimethyl sulfide complex (~8 equiv) for 20 min, which was confirmed by the disappearance of the triplet at 108 ppm and the appearance of a broad peak at ~91 ppm, and with a slight shift of the pentavalent phosphorus signal to around −19 ppm. Compounds **5** were obtained by opening the ring of **4** with l-tryptophan methyl ester (~5 equiv) overnight at rt.

For the purification of **5**, most solvents in the final reaction mixture were evaporated by a rotary evaporator; then the triphosphate analogs were immediately separated from other impurities by ion-exchange chromatography on QA52, eluting with a gradient solution of 5–200 mM ammonium bicarbonate buffer (NH_4_HCO_3_, pH 8) over 2 h. Desired fractions were then lyophilized and the *R*p/*S*p mixture of **5** was obtained in ammonium salt form. Next, the lyophilized sample was dissolved in DI water and RP-HPLC was used to separate the *R*p and *S*p stereoisomers of **5** with 0%–30% ACN linear gradients in 20 mM pH 7 TEAA buffer (in 40 min, 3 mL/min) as eluting solvent.

Further treatment of the isolated **5a** or **5b** by NH_4_OH (~17%) for 6 h at rt yielded **6a** or **6b,** respectively, along with the remaining **5a** or **5b**. Then, most solvents and ammonia of the reaction mixture of **6a** or **6b** were evaporated with a rotary evaporator at low temperature, and the residue was resolved by HPLC using 1%–21% ACN/20 mM TEAA (in 40 min at 3 mL/min) to give the pure isomer (Supplementary Figures S1 and S2).

The *R*p/*S*p mixture of d4T α-*P*-boranotriphosphates (d4TTPαB) was obtained by adding DI water into the reaction mixture of **4** and stirring overnight at rt. Then d4TTPαB *R*p/Sp isomers were separated from other impurities by ion-exchange chromatography on QA52 using the conditions described above (~38% yield by UV). For d4TTPαB *R*p and *S*p isomers, no further separation by HPLC was performed in consideration of the challenge resulting from the close peak retention times in the HPLC profile (Supplementary Figure S6).

### 3.3. LC-MS Analysis of Degradation Products from d4T Triphosphate Mimics

Each sample of d4T α-*P*-Borano-γ-*P-N*-l-tryptophanyltriphosphates was dissolved in Milli-Q DI water, and the stock solution concentration was determined by UV and then diluted to 0.03 mM in 50 μL, 4 mM Tris pH 7.4 buffer (6 vials prepared for each compound) prior to first injection and incubation at physiological relevant temperature and pH. Mixing of the sample stock solution with buffer before the first injection was completed within 20–30 s. The first injection time of sample into the Varian HPLC (Supplementary Figure S2) was referred to as *t* = 0. This injection was followed by an immediate incubation at 37 ± 0.5 °C. Samples were collected over a period of 16 days for LC-MS analysis (example chromatograms are shown in Supplementary Figures S3–S5). Either 5 or 6 µL hydrolysis mixture was injected into the Agilent 1100 Series LC-MS system and eluted at 0.3 mL/min with ACN/10 mM TEAA buffer, 0%–25% ACN in 25 min. The column (Eclipse XDB C-18, 2.1 mm × 50 mm ZORBAX, 3.5 μm) was cleaned with 80% ACN for about 10 min after each injection.

### 3.4. Spectroscopic Data

All compounds were isolated in TEA salt form. NMR experiments were performed at 2 °C unless specified otherwise. The ^1^H-NMR peaks from salt TEAA (δ ppm ~1.8 for COMe, and 3.1 and 1.2 for ethyl group at 25 °C; ~1.5 for COMe, and 2.8 and 0.9 for ethyl group at 2 °C) are not listed in the data section. NMR scans are given in Figures S7–S22.

*d4T 5′-α-P-Borano-γ-P-N-l-TrpOMe triphosphate* (**5a**). C_22_H_29_BN_4_O_13_P_3_, 661 (by LC-MS, M^−^). ^1^H-NMR (D_2_O, 500 MHz, δ ppm): 7.05 (d, *J* = 8 Hz, 1H, H-4′′), 6.99 (d, *J* = 8 Hz, 1H, H-7′′), 6.96 (s, 1H, H-6), 6.83 (s, H-2′′), 6.76 (t, *J* = 7.5 Hz, 1H, H-6′′), 6.66 (t, *J* = 7.5 Hz, 1H, H-5′′), 6.42 (s, 1H, H-1′), 5.98 (d, *J* = 5.5 Hz, 1H, H-2′), 5.37 (d, *J* = 5.5 Hz, 1H, H-3′), 4.67 (s, 1H, H-4′), 3.94 (m, 1H, H-5′), 3.80 (m, 2H, H-5′ and CH on Trp), 3.08 (s, 3H, CH_3_ from CO_2_Me), 2.91 and 2.72 (2m, 2H, CH_2_ on Trp), 1.30 (s, 3H, CH_3_-5), +0.4 to −0.3 (br, 3H, BH_3_); ^31^P-NMR (D_2_O, 202 MHz, δ ppm): 84.16 (br, 1P, α-P), −3.90 (d, *J* = 21.0 Hz, 1P, γ-P), −22.33 (t, *J* = 30.10 Hz, 1P, β-P); UV (H_2_O) λ_max_ 269 nm, ε = 1.47 × 10^4^ M^−1^·cm^−1^; HRMS found: *m*/*z* 661.1042 (for ^11^B), calcd: 661.1042.

*d4T 5′-α-P-Borano-γ-P-N-l-TrpOMe triphosphate* (**5b**). C_22_H_29_BN_4_O_13_P_3_, 661 (by LC-MS, M^−^). ^1^H-NMR (D_2_O, 500 MHz, 25 °C, δ ppm): 7.44 (d, *J* = 7.5 Hz, 1H, H-4′′), 7.32 (d, 1H, H-7′′), 7.31 (s, 1H, H-6), 7.18 (s, H-2′′), 7.07 (t, *J* = 7.5 Hz, 1H, H-6′′), 6.99 (t, *J* = 7.5 Hz, 1H, H-5′′), 6.69 (s, 1H, H-1′), 6.31 (d, *J* = 6.0 Hz, 1H, H-2′), 5.62 (d, *J* = 6.0 Hz, 1H, H-3′), 4.90 (s, 1H, H-4′), 4.09 (3m, 3H, H-5′ and CH on Trp), 3.39 (s, 3H, CH_3_ from CO_2_Me), 3.27 and 3.07 (2m, 2H, CH_2_ on Trp), 1.66 (s, 3H, CH_3_-5), +0.7 to −0.1 (br, 3H, BH_3_); ^31^P-NMR (D_2_O, 202 MHz, δ ppm): 84.74 (br, 1P, α-P), −3.91 (d, *J* = 20.81 Hz, 1P, γ-P), −22.26 (d, *J* = 25.65 Hz, 1P, β-P); UV (H_2_O) λ_max_ 269 nm, ε = 1.47 × 10^4^ M^−1^·cm^−1^; HRMS found: *m*/*z* 661.1039 (for ^11^B), calcd: 661.1042.

*d4T 5′-α-P-Borano-γ-P-N-l-TrpNH_2_ triphosphate* (**6a**). C_21_H_28_BN_5_O_12_P_3_, 646 (by LC-MS, M^−^). ^1^H-NMR (D_2_O, 500 MHz, δ ppm): 7.21 (d, *J* = 8 Hz, 1H, H-4′′), 7.02 (d, *J* = 8 Hz, 1H, H-7′′), 6.95 (s, 1H, H-6), 6.89 (s, H-2′′), 6.77 (t, *J* = 7.5 Hz, 1H, H-6′′), 6.68 (t, *J* = 7.5 Hz, 1H, H-5′′), 6.47 (s, 1H, H-1′), 5.96 (d, *J* = 5.5 Hz, 1H, H-2′), 5.37 (d, *J* = 5.5 Hz, 1H, H-3′), 4.62 (s, 1H, H-4′), 3.84 and 3.73 (m, 2H, H-5′), 3.67 (m, H, CH on Trp), 3.00 and 2.79 (2m, 2H, CH_2_ on Trp), 1.33 (s, 3H, CH_3_-5), +0.4 to −0.4 (br, 3H, BH_3_); ^31^P-NMR (D_2_O, 202 MHz, δ ppm): 84.78 (br, 1P, α-P), −3.78 (d, *J* = 21.05 Hz, 1P, γ-P), −22.18 (t, 1P, β-P); UV (H_2_O) λ_max_ 269 nm, ε = 1.47 × 10^4^ M^−1^·cm^−1^; HRMS found: *m*/*z* 646.1066 (for ^11^B), calcd: 646.1046.

*d4T 5′-α-P-Borano-γ-P-N-l-TrpNH_2_ triphosphate* (**6b**). C_21_H_28_BN_5_O_12_P_3_, 646 (by LC-MS, M^−^). ^1^H-NMR (D_2_O, water presaturated, 500 MHz, 25 °C, δ ppm): 7.53 (d, *J* = 8 Hz, 1H, H-4′′), 7.31 (d, *J* = 8 Hz, 1H, H-7′′), 7.24 (s, 1H, H-6), 7.20 (s, H-2′′), 7.06 (t, *J* = 7.5 Hz, 1H, H-6′′), 6.97 (t, *J* = 7.5 Hz, 1H, H-5′′), 6.93 (s, 1H, H-1′), 6.31 (d, *J* = 6.0 Hz, 1H, H-2′), 5.59 (d, *J* = 6.0 Hz, 1H, H-3′), 4.87 (s, 1H, H-4′), 3.99 (m, 3H, H-5′ and CH on Trp), 3.27 and 3.01 (2m, 2H, CH_2_ on Trp), 1.65 (s, 3H, CH_3_-5), +0.7 to −0.1 (br, 3H, BH_3_); ^31^P-NMR (D_2_O, 202 MHz, δ ppm): 85.00 (br, 1P, α-P), −3.55 (br, 1P, γ-P), −21.83 (br, 1P, β-P); UV (H_2_O) λ_max_ 269 nm, ε = 1.47 × 10^4^ M^−1^·cm^−1^; HRMS found: *m*/*z* 646.1046 (for ^11^B), calcd: 646.1046.

*d4T* *α-P-Boranotriphosphate Rp* (**7a**). C_10_H_17_BN_2_O_12_P_3_, 461 (by LC-MS, M^−^). ^1^H-NMR (D_2_O, 400 MHz, 25 °C, δ ppm): 7.39 (s, 1H, H-6), 6.75 (s, 1H, H-1′), 6.39 (d, *J* = 5.2 Hz, 1H, H-2′), 5.77 (d, *J* = 5.2 Hz, 1H, H-3′), 4.94 (s, 1H, H-4′), 4.08 and 3.96 (m, 2H, H-5′), 1.75 (s, 3H, CH_3_-5), +0.9 to −0.3 (br, 3H, BH_3_); ^31^P-NMR (D_2_O, 202 MHz, 25 °C, δ ppm): 84.30 (br, 1P, α-P), −5.45 (br, 1P, γ-P), −21.45 (br, 1P, β-P); UV (H_2_O) λ_max_ 266 nm.

*d4T* *α-P-Boranotriphosphate Sp* (**7b**). C_10_H_17_BN_2_O_12_P_3_, 461 (by LC-MS, M^−^). ^1^H-NMR (D_2_O, 500 MHz, 25 °C, δ ppm): 7.47 (s, 1H, H-6), 6.81 (s, 1H, H-1′), 6.39 (d, 1H, H-2′), 5.81 (d, 1H, H-3′), 4.99 (s, 1H, H-4′), 4.17 and 4.03 (m, 2H, H-5′), 1.80 (s, 3H, CH_3_-5), +0.7 to −0.2 (br, 3H, BH_3_); ^31^P-NMR (D_2_O, 162 MHz, 25 °C, δ ppm): 83.83 (br, 1P, α-P), −5.93 (br, 1P, γ-P), −21.34 (br, 1P, β-P); UV (H_2_O) λ_max_ 266 nm.

## 4. Conclusions

To summarize, one pot synthesis of d4T α-*P*-boranotriphosphate (conjugated with a tryptophan methyl ester or amide) analogs was achieved via a modified cyclic trimetaphosphate approach. Because of reasonable yields and convenience, this approach could be used as a general method to obtain other nucleoside γ-*P*-*N*-substituted boranotriphosphate mimics (as potential NRTI prodrugs). The hydrophobic borane group should increase the mimic stability towards cellular enzymes, e.g., nucleases. *R*p and *S*p stereoisomers of the title pronucleotides were purified via a combination of chromatographic methods. Parallel degradation pathways imparted by structural modifications on the tryptophan moiety allow the selective chemical release of different nucleotides. Specifically, after incubation (at pH 7.4) of d4T 5′-α-*P*-borano-γ-*P*-*N*-l-TrpOMe triphosphates (**5a**, **5b**), the main hydrolysis products generated were the corresponding triphosphates (d4TTPαB, via intermolecular attack by water on the γ-P). By contrast, the amide mimics (**6a**, **6b**) mainly degraded to the corresponding diphosphates (d4TDPαB, via intramolecular nucleophilic attack by the amino group of the amide on the γ-P, whereby degradation was facilitated by the amide group, such that compounds **6** are less stable than **5**). In all cases, d4T H-phosphonate **8** was produced as the secondary degradation product over time. We propose that compound **8** is generated via a protonation-promoted intramolecular reduction followed by a second step nucleophilic attack. We believe this report is of significance because at one time it was unclear whether nucleoside boranotriphosphoramidate stereoisomers could be resolved or sufficiently stable. The stabilities of the title compounds (half-lives 5–36 days) support the rationale to make similar compounds for further investigations of unique properties imparted by the isoelectronic substitution of a borane group (BH_3_) for one of the nonbridging oxygens in the α-phosphate moiety. Our ddN triphosphoramidate approach might also provide useful information in the pronucleotide-based drug design for a controlled drug release of target nucleotides. We propose that once these ddN triphosphoramidate prodrugs have penetrated into target cells, they might be activated enzymatically and/or chemically to the corresponding nucleoside triphosphates.

## Supplementary Materials

Supplementary materials can be accessed at: http://www.mdpi.com/1420-3049/20/10/18808/s1.
