# Supplementary material for: Synthesis, Hydrolysis, and Protonation-Promoted Intramolecular Reductive Breakdown of Potential NRTIs: Stavudine α-P-Borano-γ-P-N-l-tryptophanyltriphosphates
Source: Molecules. 2015 Oct 16;20(10):18808–26. doi: 10.3390/molecules201018808 (PMC6332514; doi:10.3390/molecules201018808)
Supplement: Supplementary file 1 [file molecules-20-18808-s001.pdf]

Supporting Materials

Synthesis, Hydrolysis, and Protonation-Promoted Intramolecular Reductive Breakdown of Potential NRTIs: Stavudine α-*P*-Borano-γ-*P*-*N*-L-tryptophanyltriphosphates

Zhihong Xu * and Barbara Ramsay Shaw

Department of Chemistry, Duke University, Durham, NC 27708, USA;
E-Mail: barbara.r.shaw@duke.edu

* Author to whom correspondence should be addressed; E-Mail: zhihong.xu@duke.edu or zxu@live.unc.edu; Tel.: +1-919-966-5908; Fax: +1-919-660-1605.

Detailed HPLC Chromatograms and NMR Spectra. Example LC-MS Analyses of Degradation Products

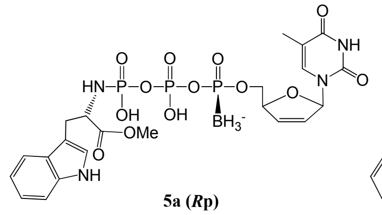

5a (*R*p)

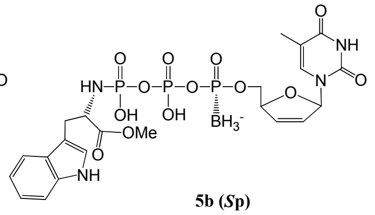

5b (*S*p)

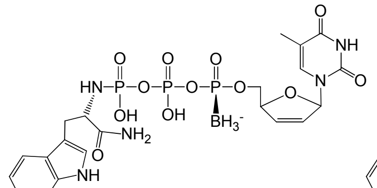

6a (*R*p)

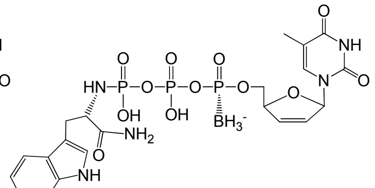

6b (*S*p)

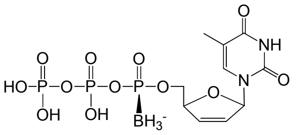

7a (d4TTPB *R*p)

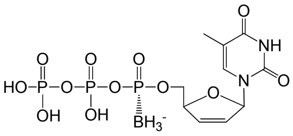

7b (d4TTPB *S*p)

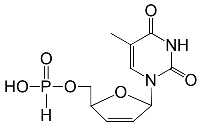

8 (d4TH-P)

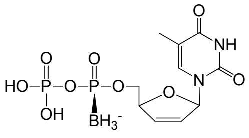

9a (d4TDPB *R*p)

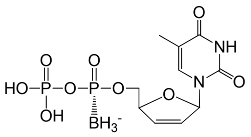

9b (d4TDPB *S*p)

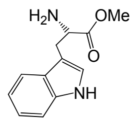

10 (TrpOMe)

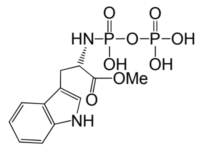

11 (TrpOMeDP)

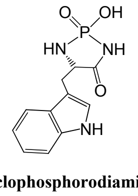

12 (Trpcyclophosphorodiamidate)

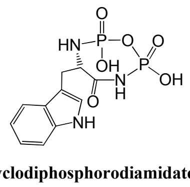

13 (Trpcyclodiphosphorodiamidate)

Chart S1. Structures of d4T α-*P*-borano-γ-*P*-*N*-L-tryptophanyltriphosphates and major degradation products in Tris buffer.

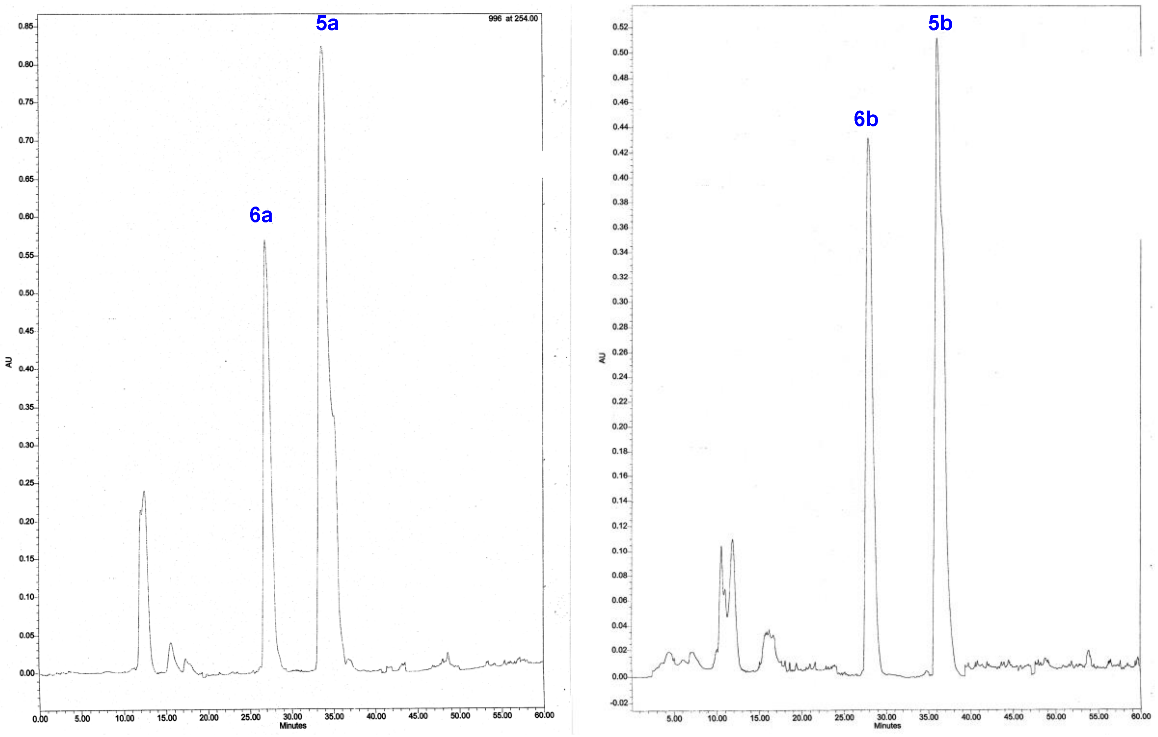

Figure S1. Isolation of **6a** and **6b** using HPLC after 17% NH_4OH treatment of the corresponding isomer of **5** at rt for 6 h. Waters™ (Milford, MA, USA) Delta 600E system (XTerra Prep RP-18 Column, 5 µm, 10 mm × 150 mm, UV detection), 0%–20% B in 40 min, 20%–90% B for an additional 10 min at 3 mL/min (solvent A: 1% acetonitrile (ACN) in 20 mM TEAA, solvent B: 100% ACN).

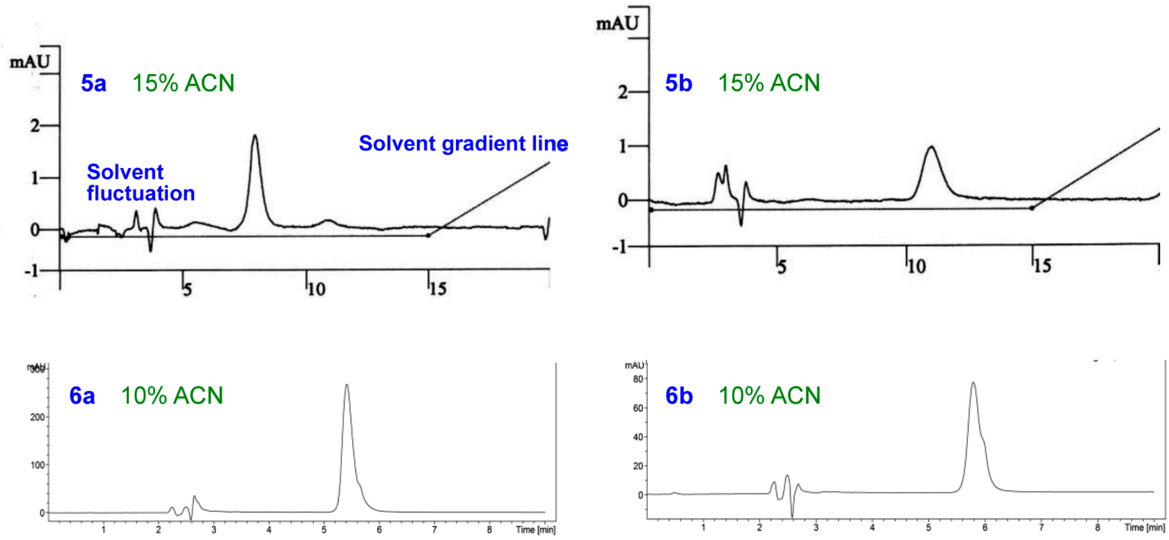

Figure S2. HPLC chromatograms of purified compounds **5a**, **5b**, **6a**, and **6b** at $t = 0$. Varian (Palo Alto, CA, USA) Star #1, UV detection at 254 nM. Column: Waters Delta-Pak™ C18, 15 µm, 3.9 mm × 300 mm. Tested at 1 mL/min with isocratic condition of 15% or 10% ACN in 10 mM TEAA (pH 7) before column cleaning.

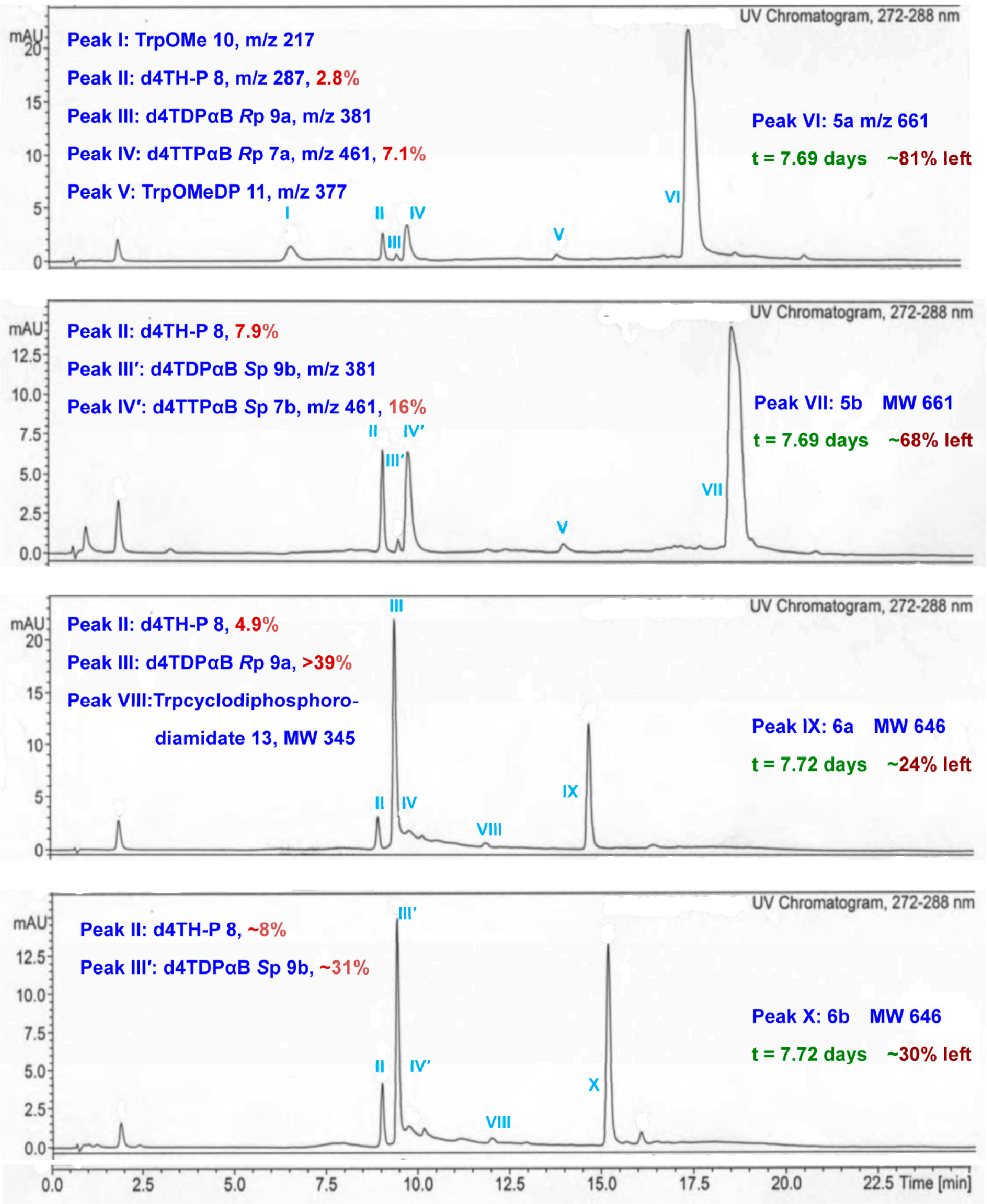

Figure S3. LC-MS analyses of 0.03 mM d4T boranotriphosphate analogs **5a**, **5b**, **6a** and **6b** in 4 mM, pH 7.4 Tris buffer at 37 ± 0.5 °C, after ~7.7 day incubation. LC-MS (Agilent, Santa Clara, CA, USA) condition: Eluted at 0.3 mL/min with 0%–25% B in 25 min (Solvent A: 10 mM pH 7 TEAA; Solvent B: ACN). Column: Eclipse XDB C-18, 2.1 × 50 mm ZORBAX, 3.5 µm. UV detection at 272–288 nm. MS negative ion detection.

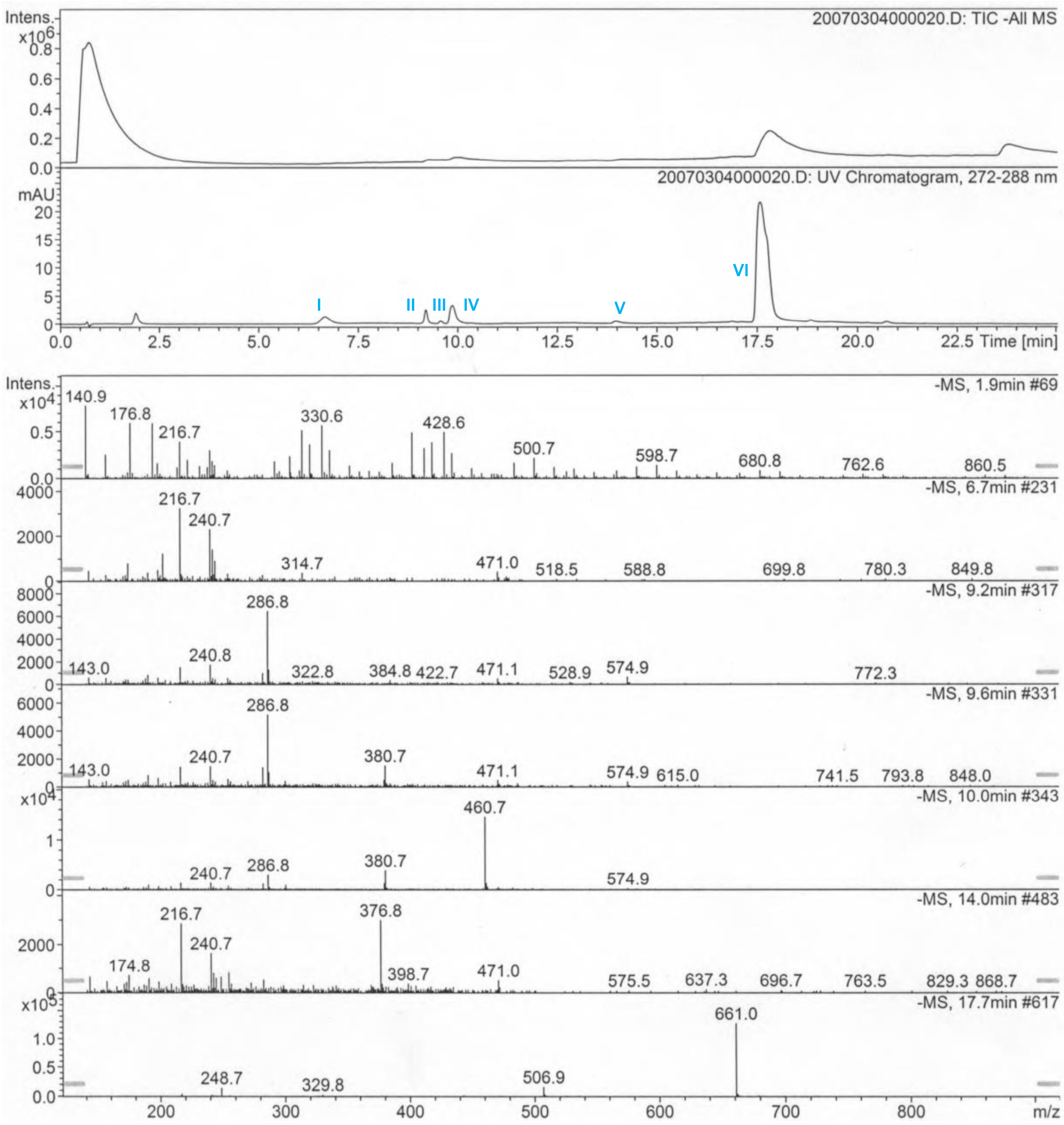

Figure S4. LC-MS analysis of 0.03 mM d4T boranotriphosphate analog **5a** in 4 mM, pH 7.4 Tris buffer at 37 ± 0.5 °C, after ~7.7 day incubation. LC-MS condition: as described in Figure S3.

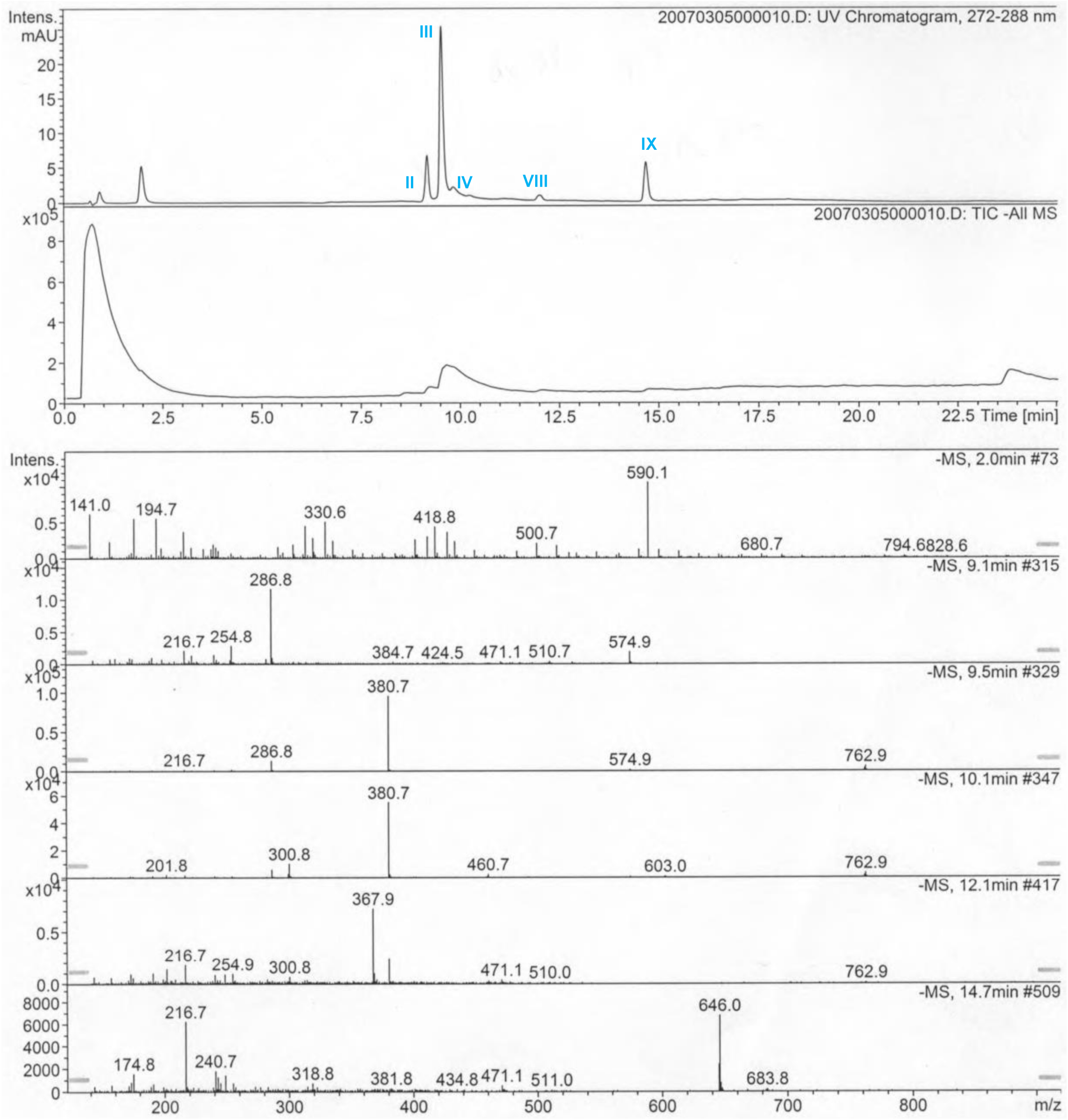

Figure S5. LC-MS analysis of 0.03 mM d4T boranotriphosphate analog **6a** in 4 mM, pH 7.4 Tris buffer at 37 ± 0.5 °C, after 16 day incubation. LC-MS condition: as described in Figure S3.

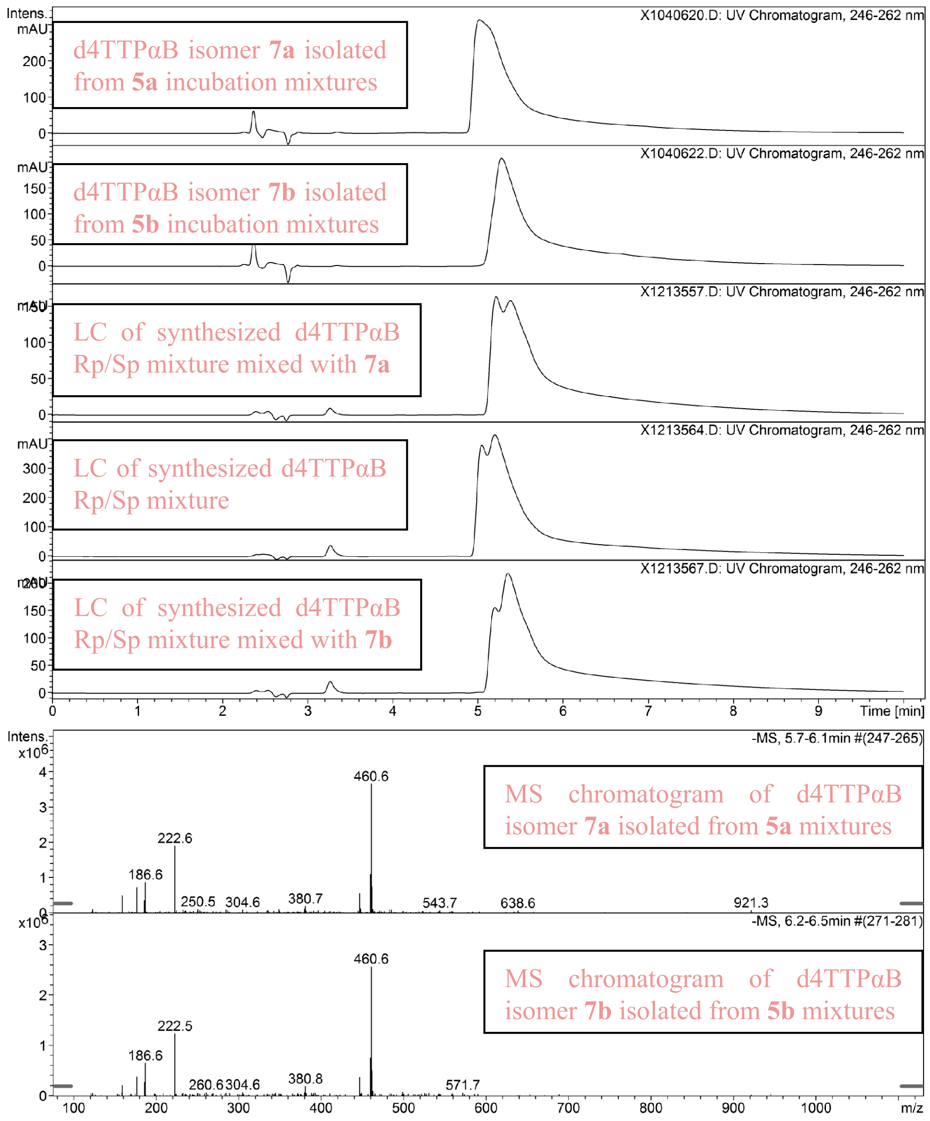

Figure S6. LC-MS verification of stereo specificity of the d4TTPαB isomers degraded from **5a** and **5b** incubation mixtures.

Incubation mixtures were the pools of the respective incubation solutions over 0.5–16 days at 37 ± 0.5 °C. LC-MS condition: Eluted at 40 µL/min with isocratic 8% B (solvent A: 5% ACN in 20 mM TEAA, solvent B: 90% ACN in H_2O). Column: Polaris 3 C-18 A 150 × 1.0 mm. UV detection at 190–400 nm. MS negative ion detection. The retention times of the two isolated isomers **7a** and **7b** were very close. As the addition of **7a** to the synthesized d4TTPαB *R*p/*S*p mixture (wherein the first and the second eluted peaks were assigned as *R*p and *S*p isomers, respectively) increased the *R*p isomer peak area, whereas **7b** increased the Sp isomer peak area, **7a** was considered to be the *R*p isomer and **7b** would be the *S*p isomer.

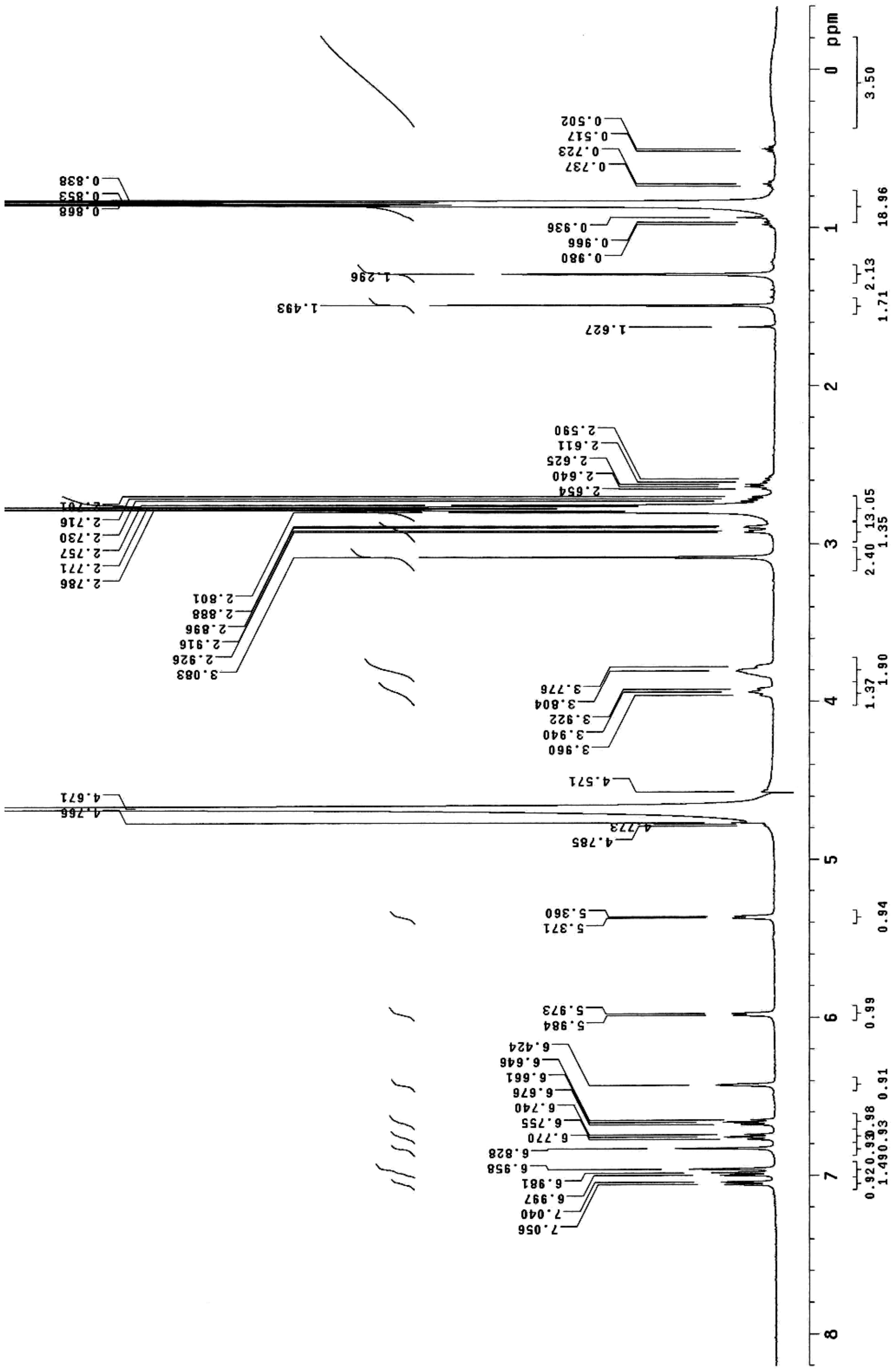

Figure S7. 5a ^{1}H-NMR in D_2O 500 MHz 2 °C.

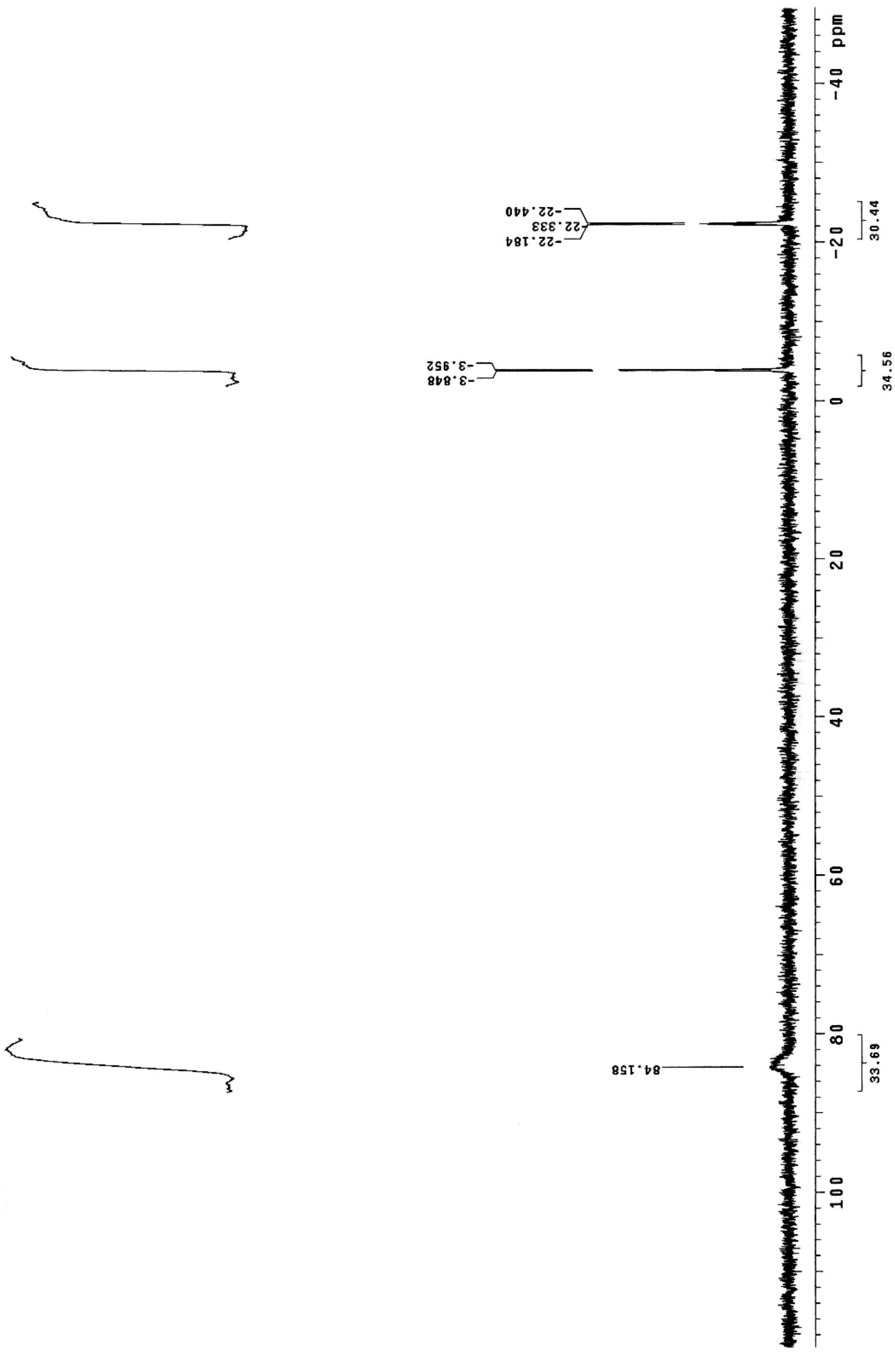

Figure S8. 5a ^{31}P-NMR in D_2O 202 MHz 2 °C.

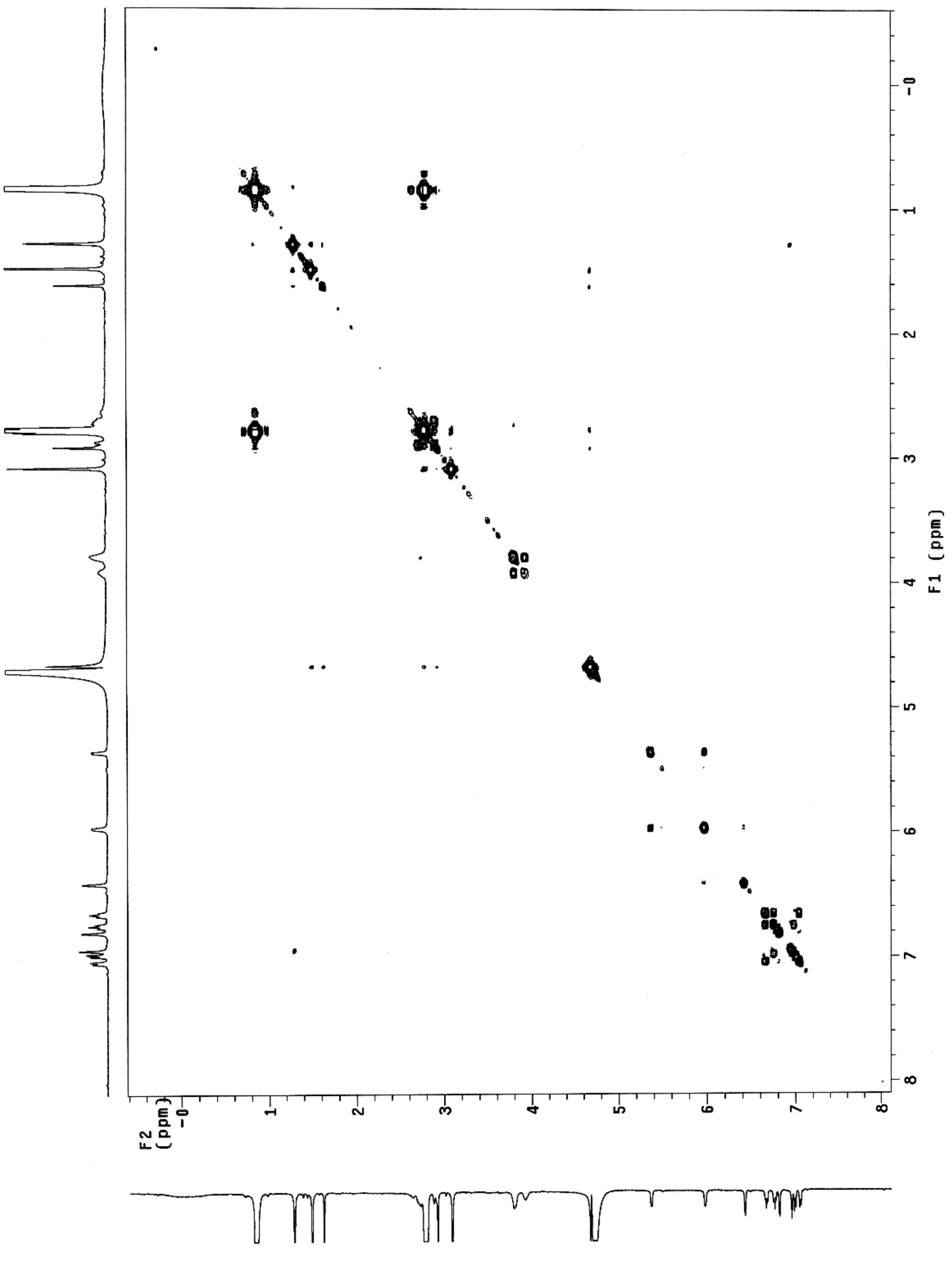

Figure S9. 5a COSY in D_2O 500 MHz 2 °C.

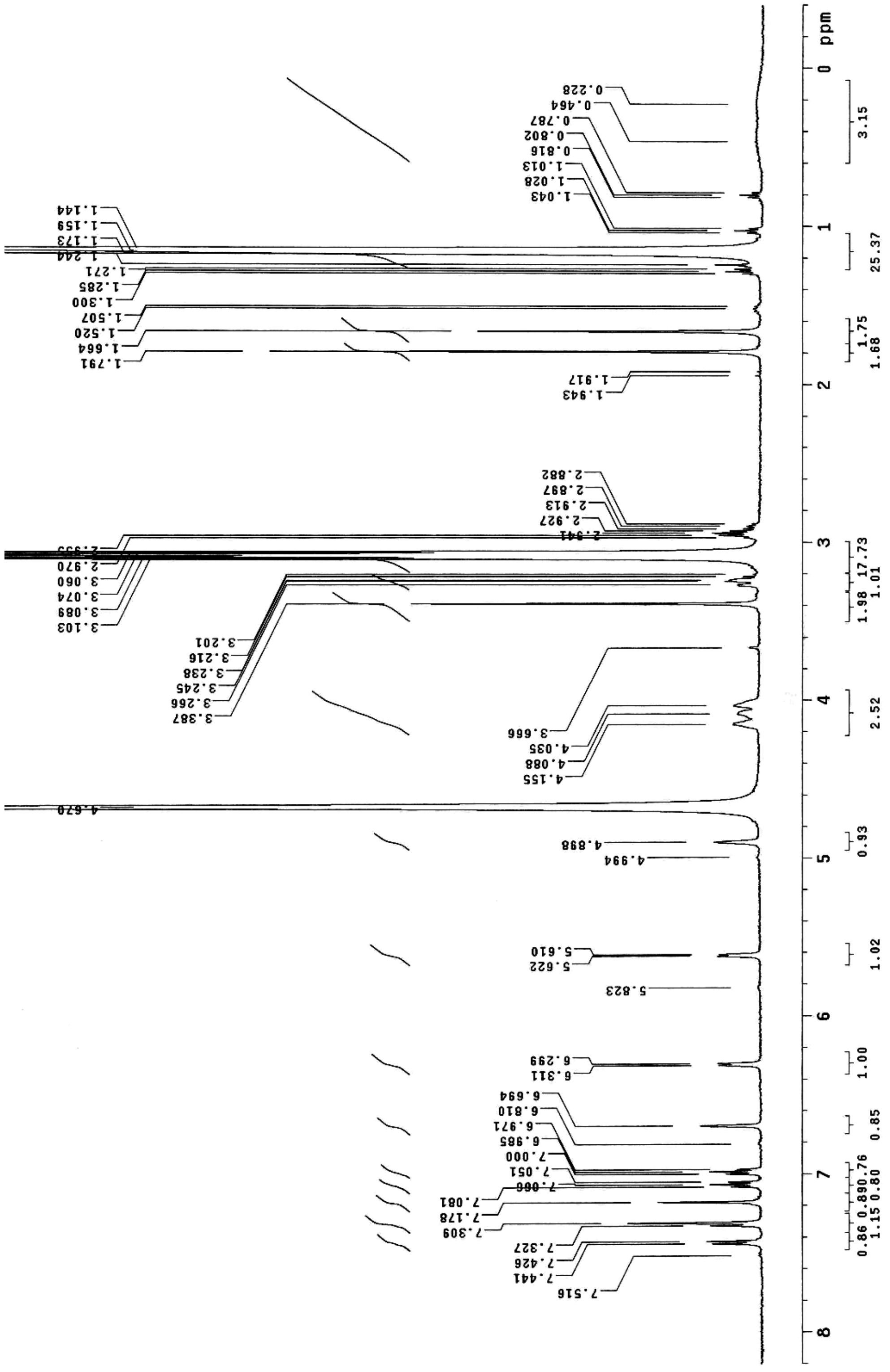

Figure S10. 5b ^{1}H-NMR in D_2O 500 MHz 25 °C.

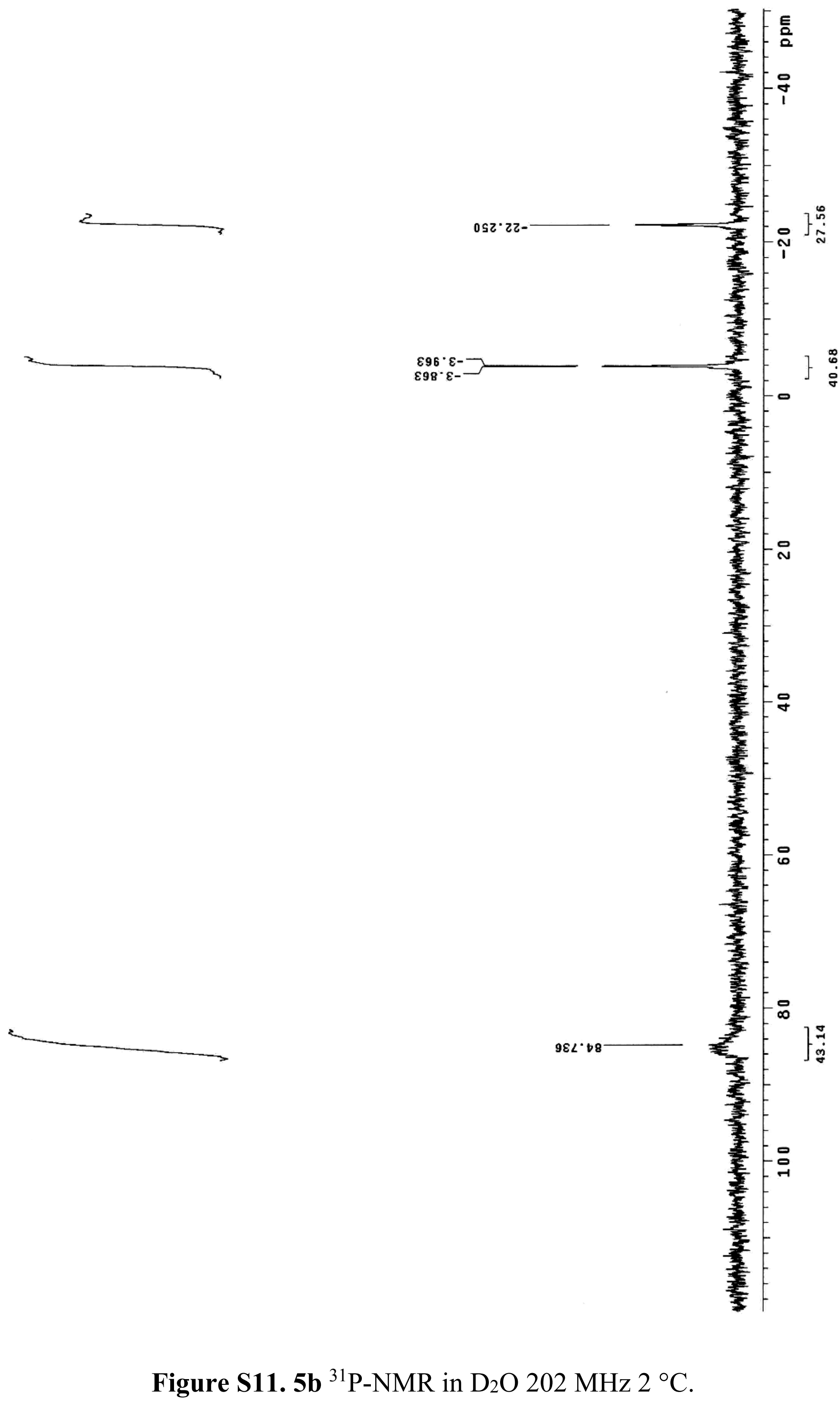

Figure S11. 5b ^{31}P-NMR in D_2O 202 MHz 2 °C.

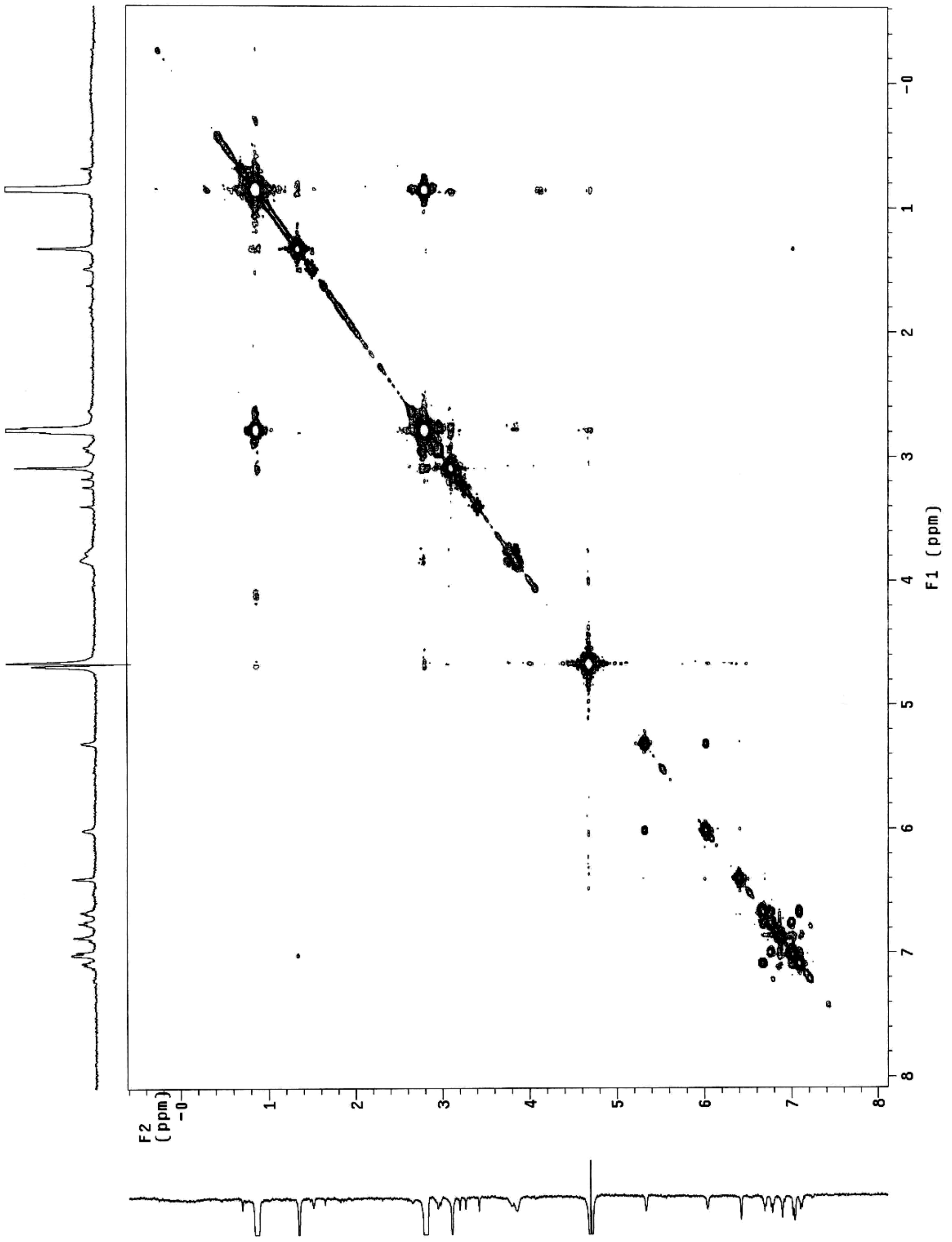

Figure S12. 5b COSY in D_2O 500 MHz 2 °C.

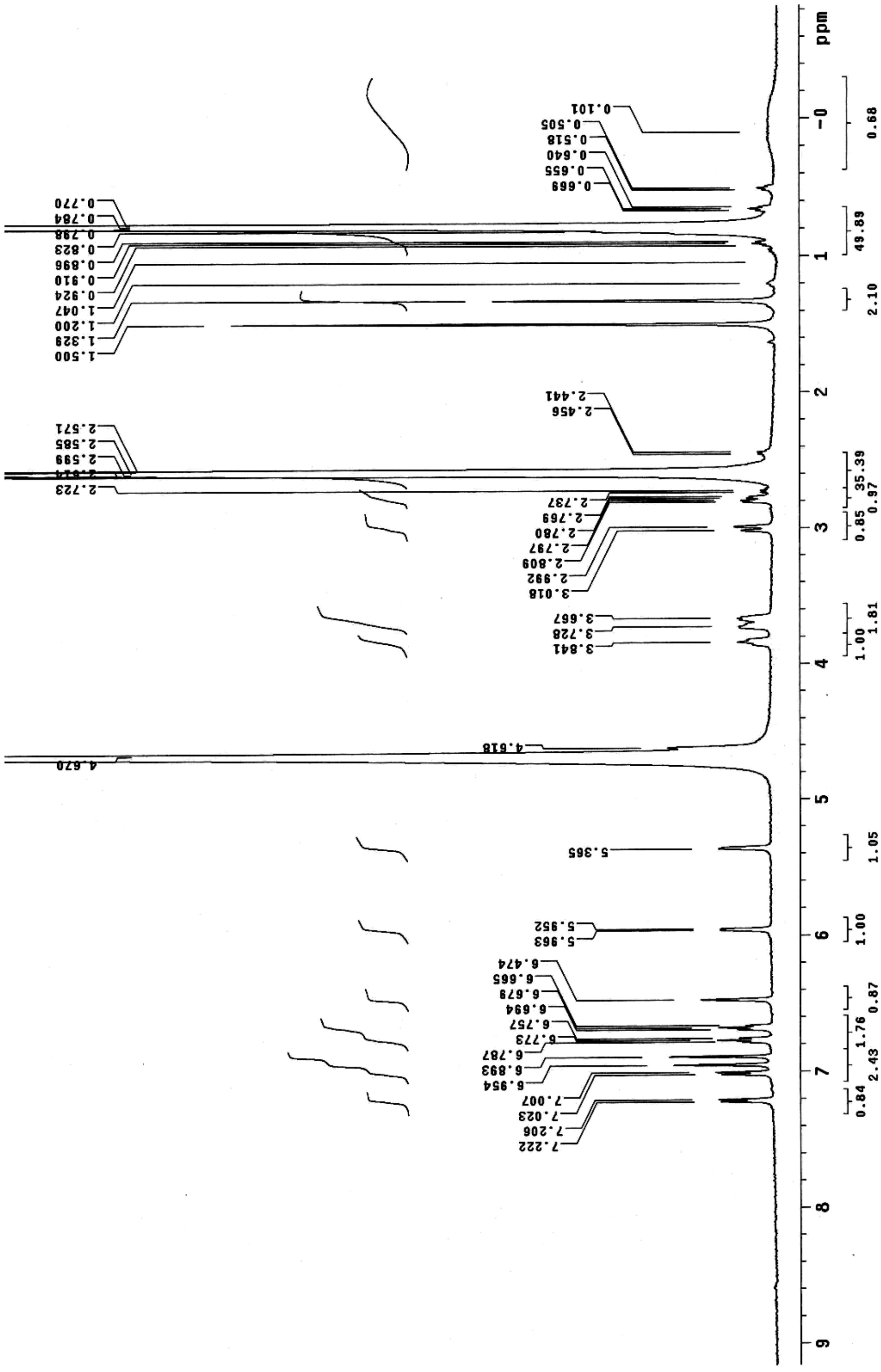

Figure S13. 6a ^{1}H-NMR in D_2O 500 MHz 2 °C.

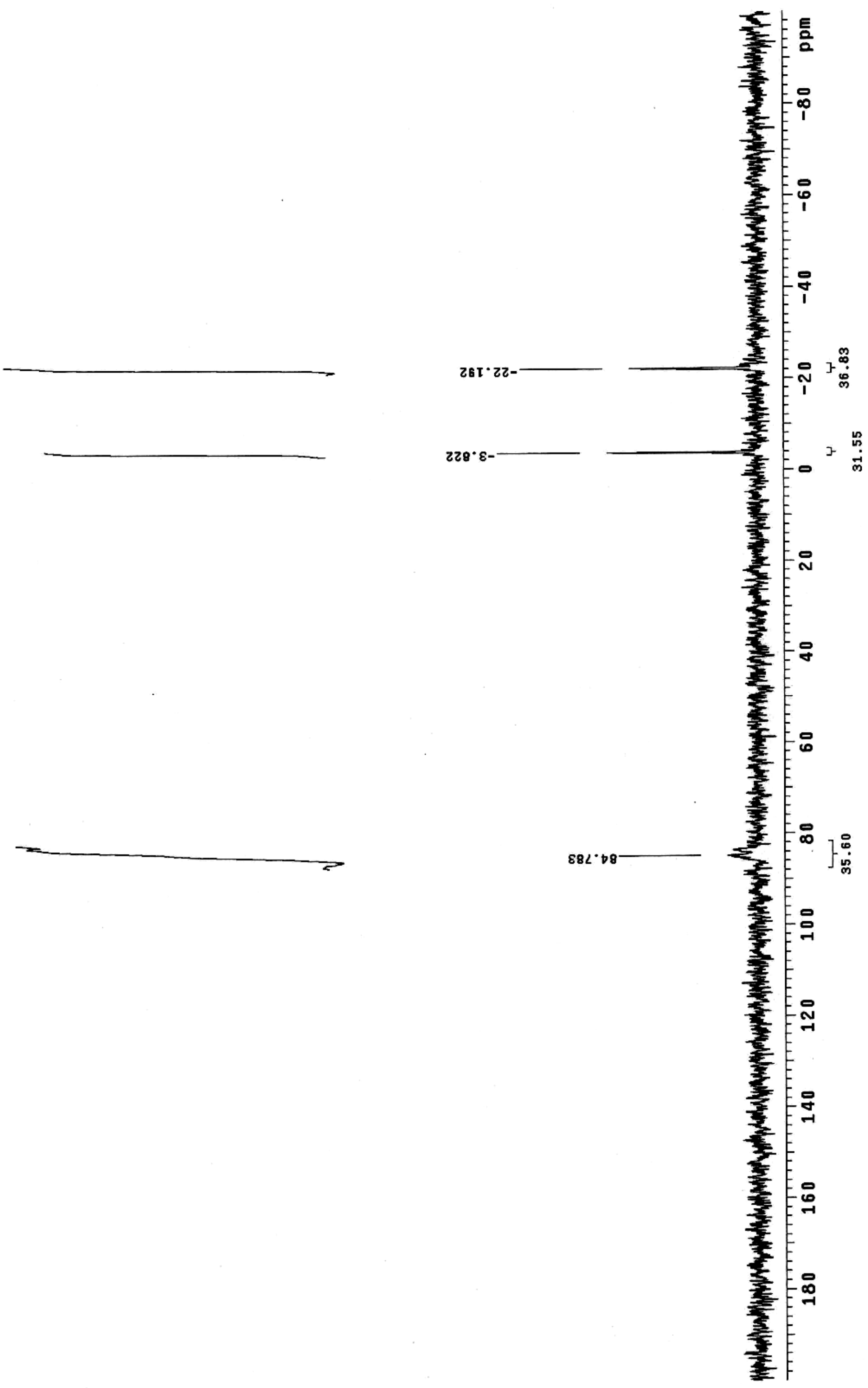

Figure S14. 6a ^{31}P-NMR in D_2O 202 MHz 2 °C.

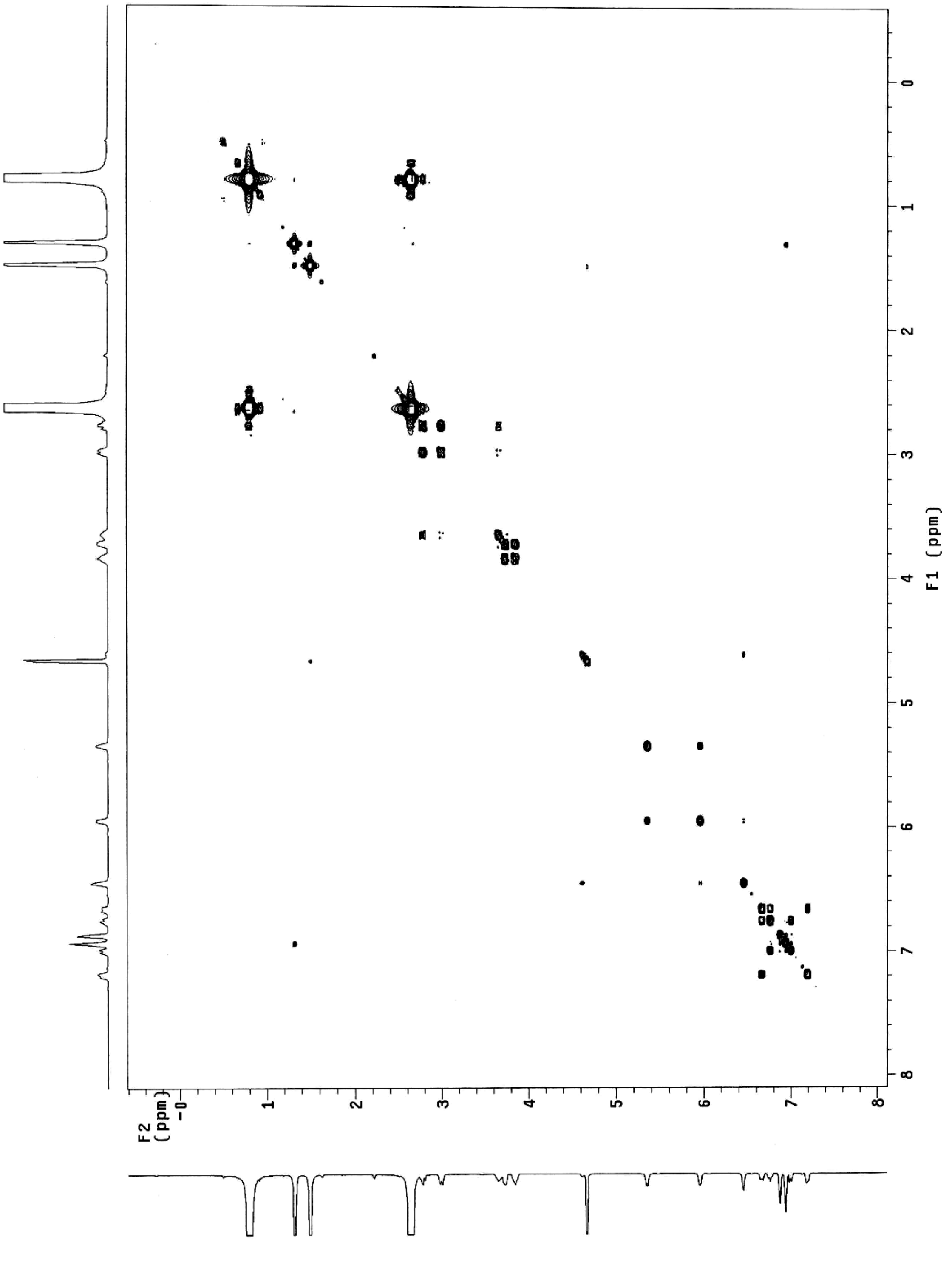

Figure S15. 6a COSY in D_2O 500 MHz 2 °C.

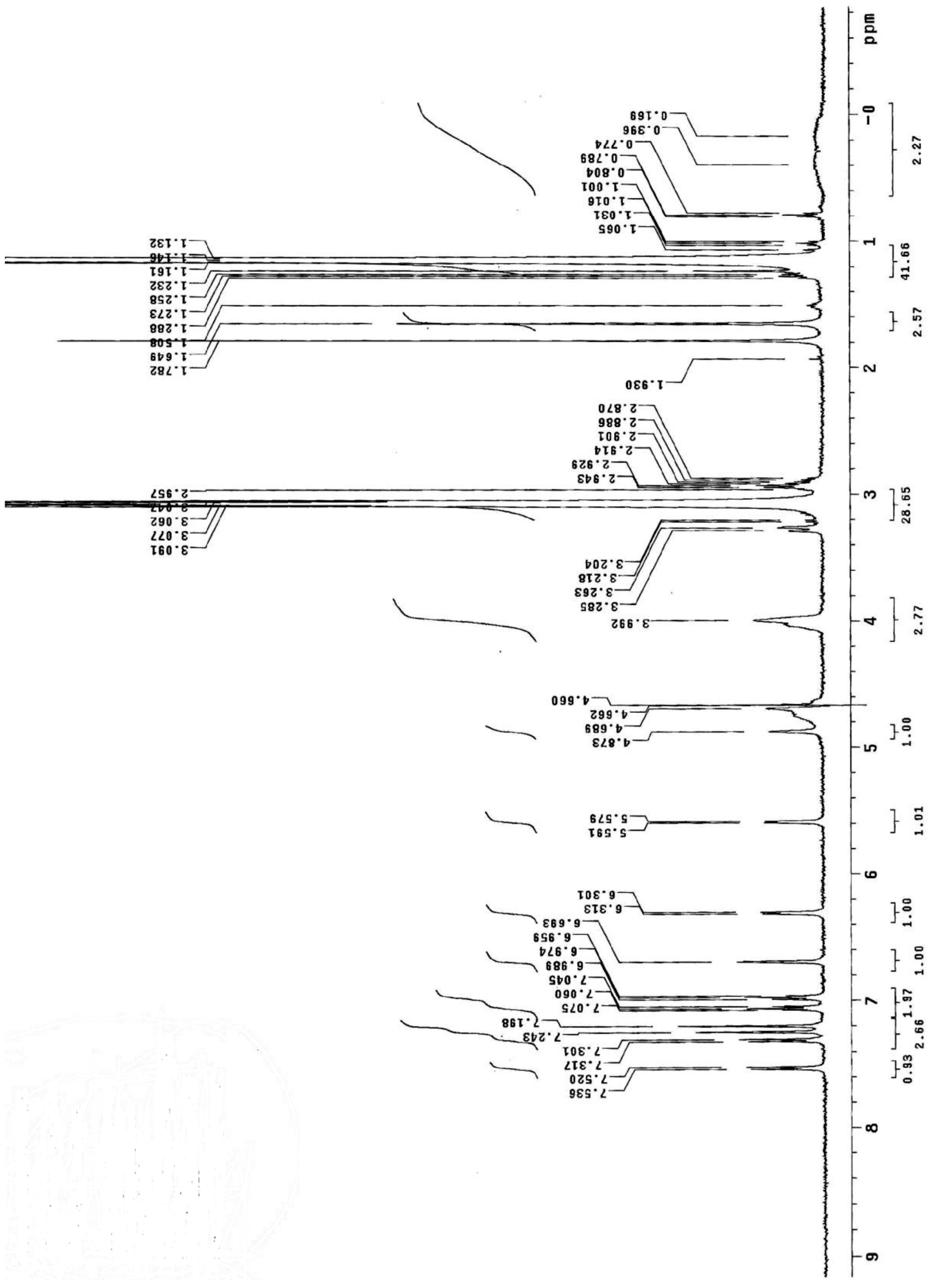

Figure S16. 6b ^{1}H-NMR in D_2O 500 MHz 25 °C Water Presat.

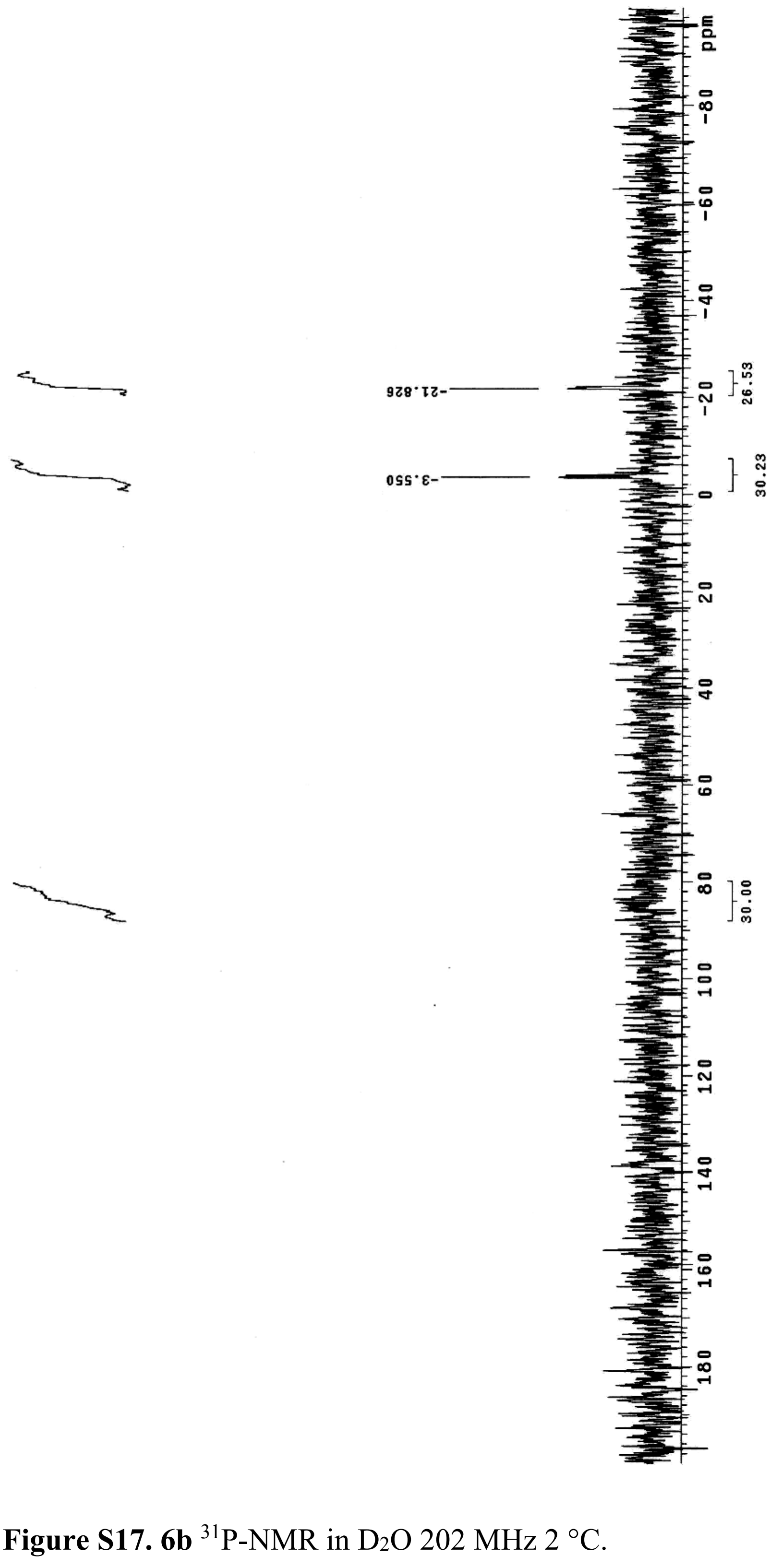

Figure S17. 6b ^{31}P-NMR in D_2O 202 MHz 2 °C.

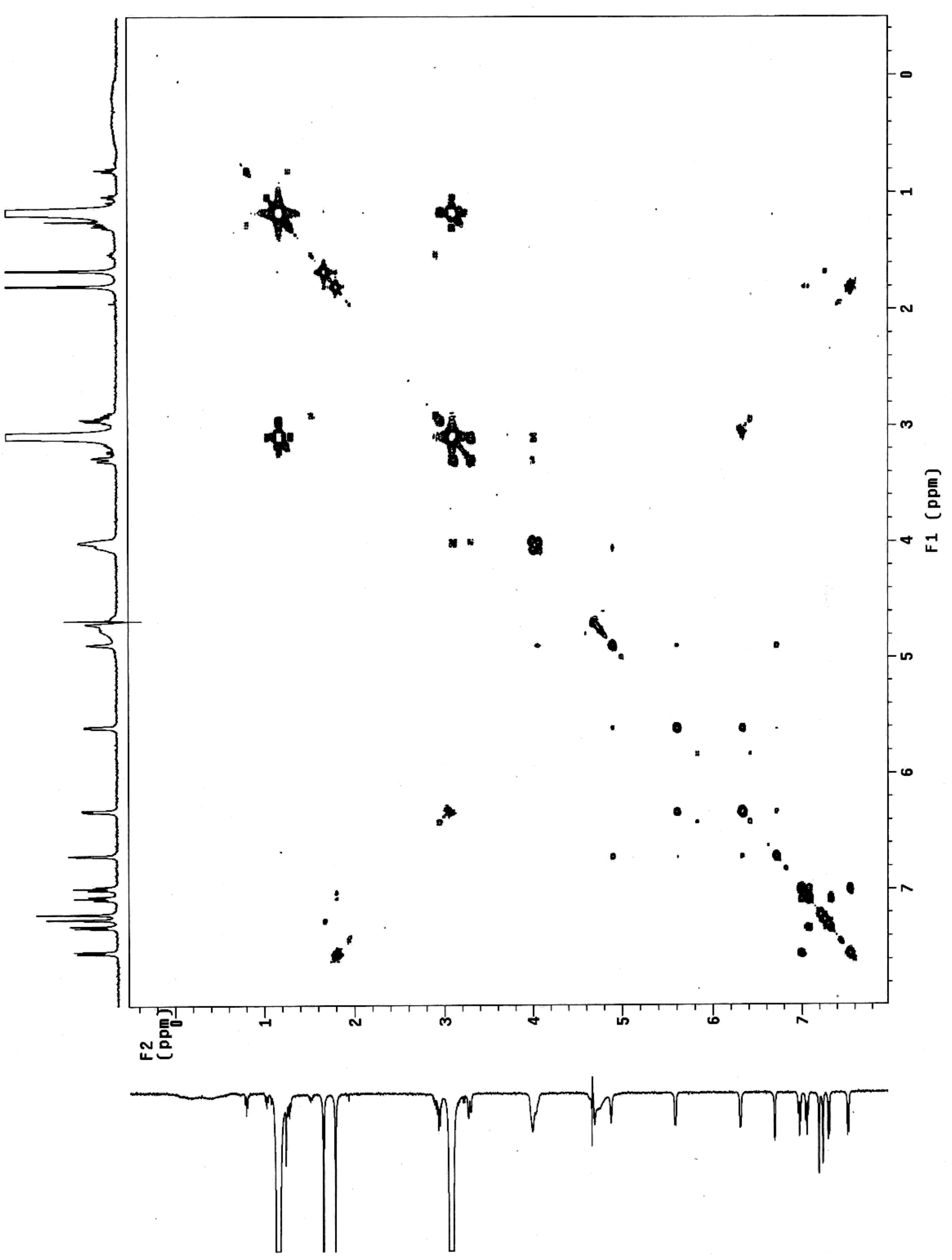

Figure S18. 6b COSY in D_2O 500 MHz 25 °C.

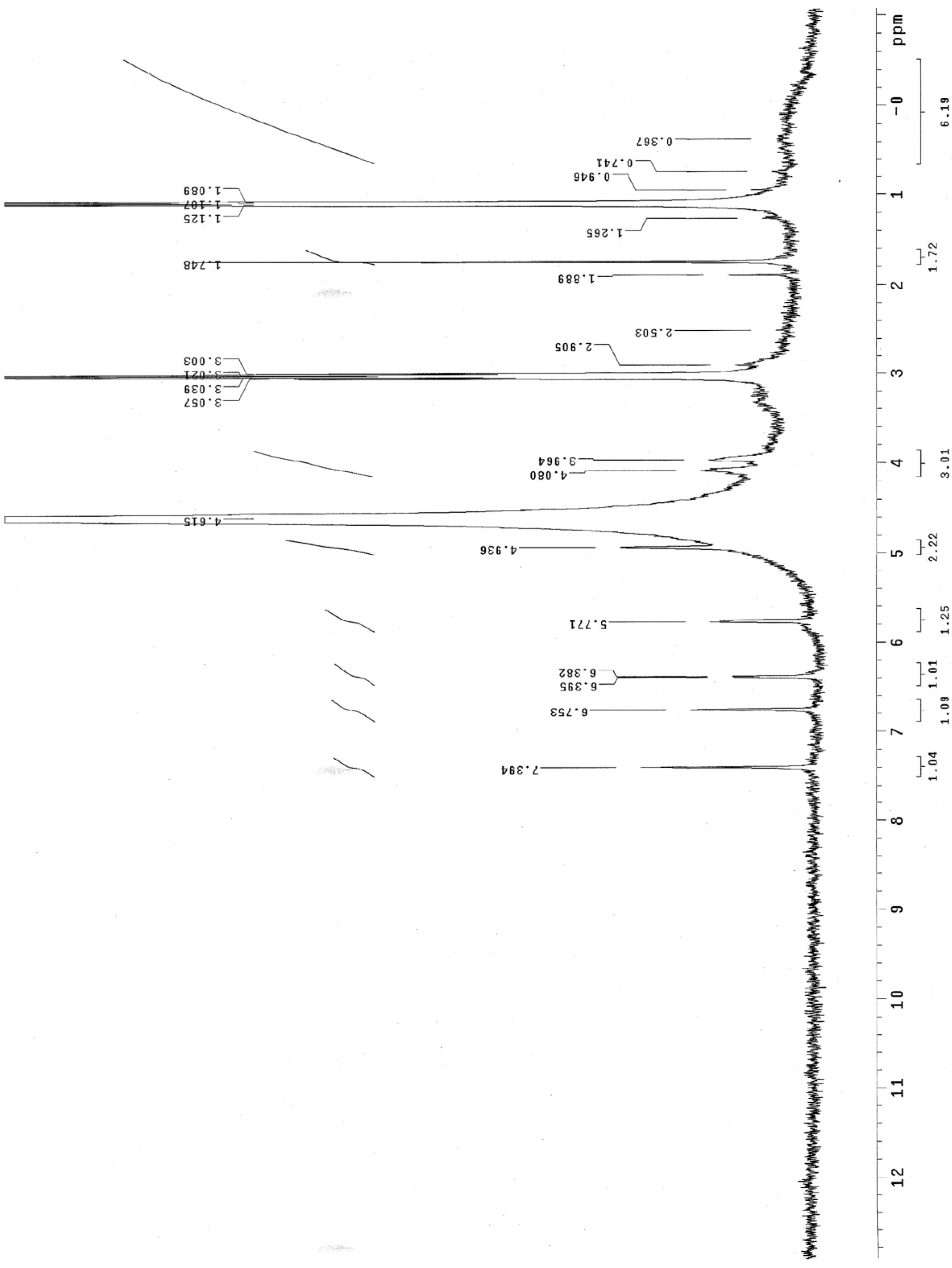

Figure S19. d4TTPαB *R*p ^{1}H-NMR in D_2O 400 MHz 25 °C.

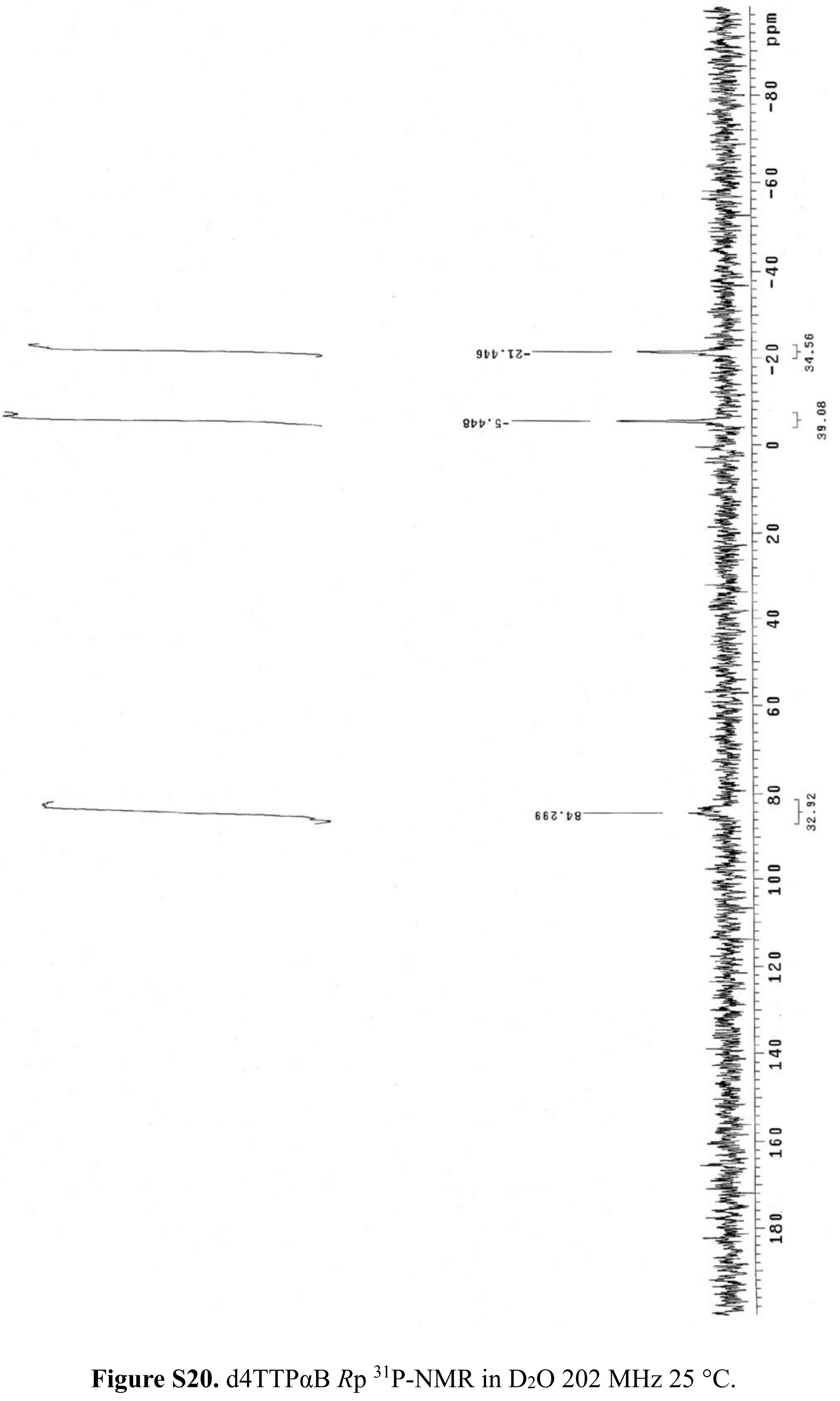

Figure S20. d4TTPαB *R*p ^{31}P-NMR in D_2O 202 MHz 25 °C.

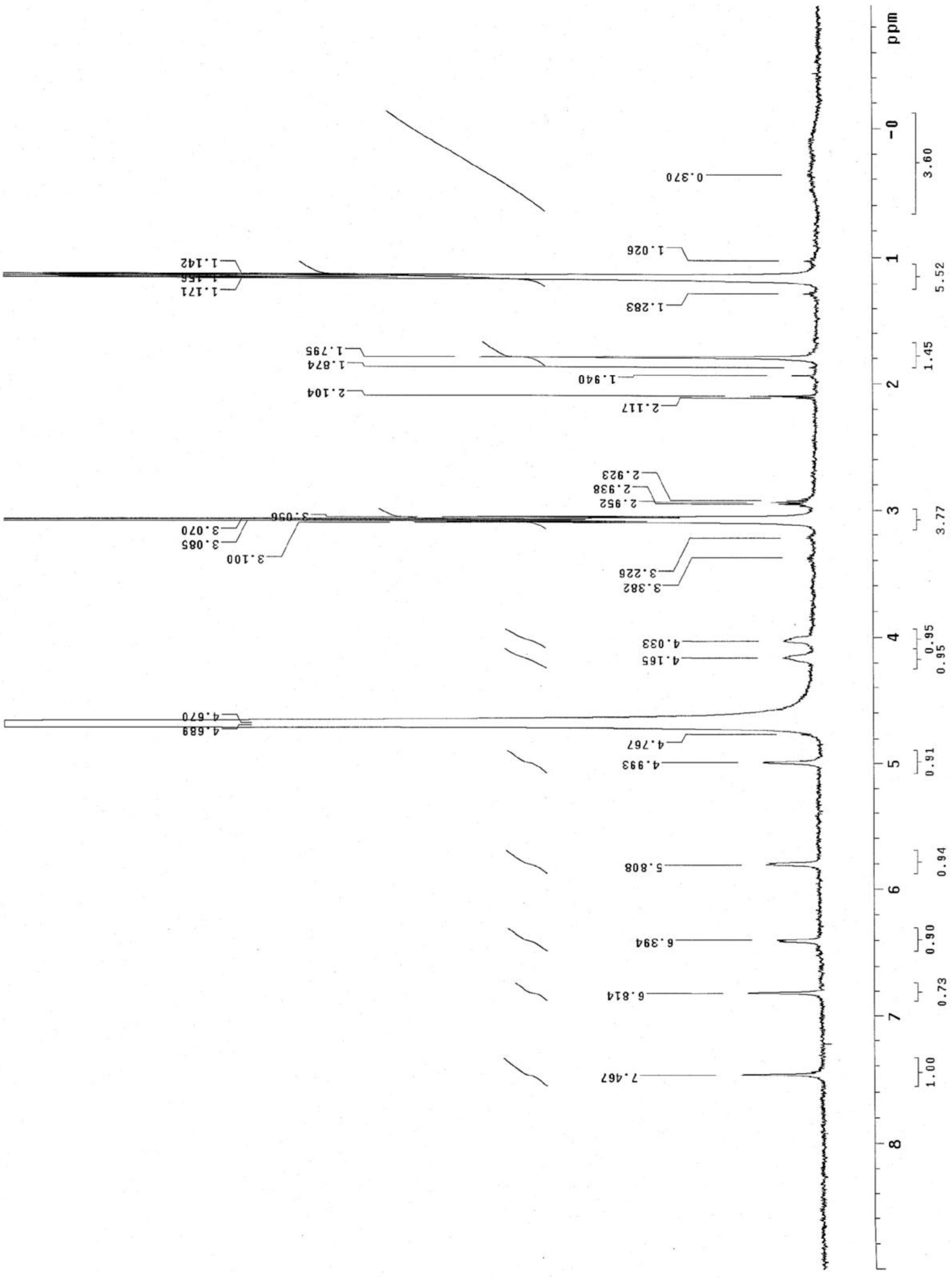

Figure S21. d4TTPαB *S*p ^{1}H-NMR in D_2O 500 MHz 25 °C.

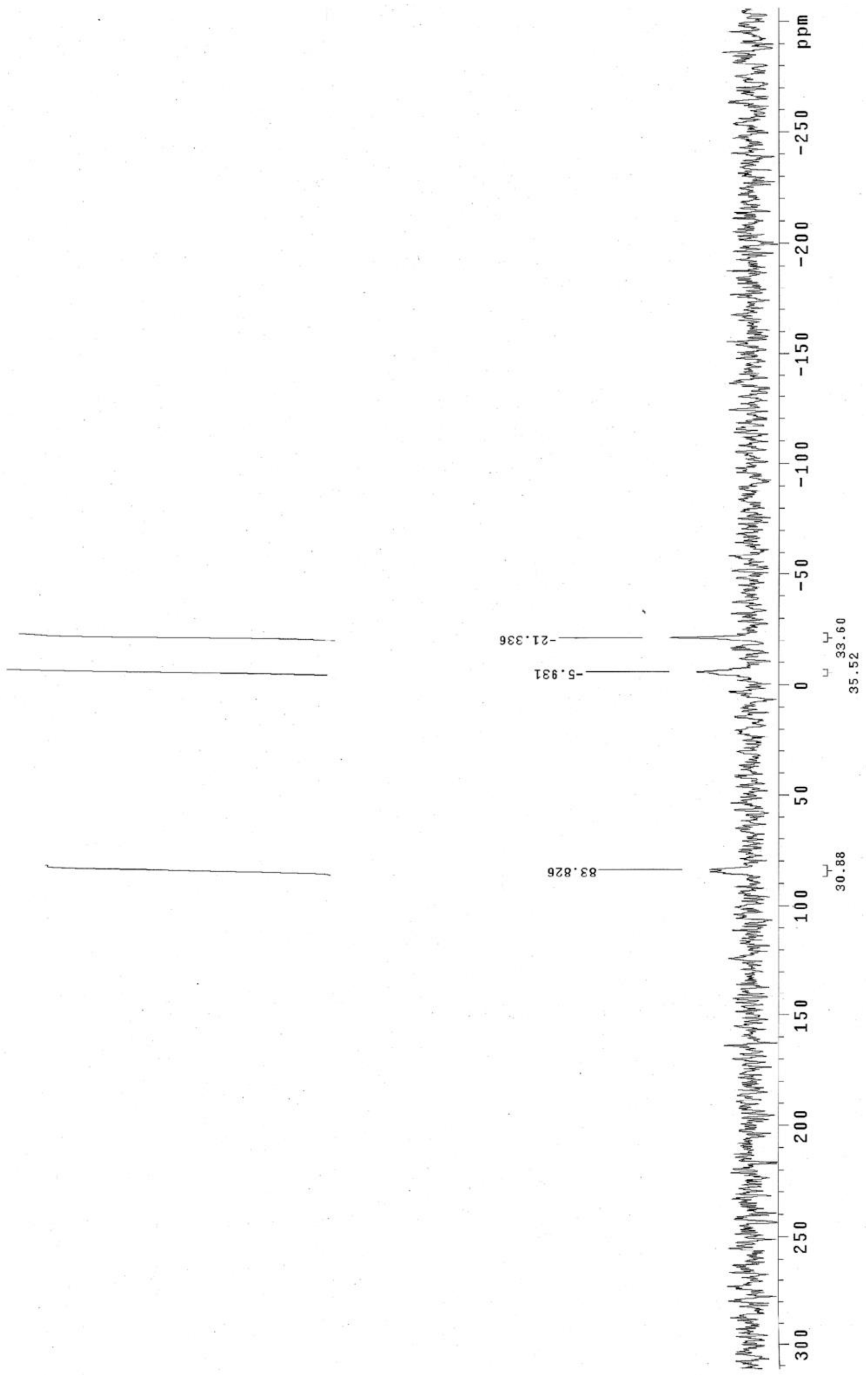

Figure S22. d4TTPαB *S*p ^{31}P-NMR in D_2O 162 MHz 25 °C.
